# Diagnostic circulating biomarkers to detect vision‐threatening diabetic retinopathy: Potential screening tool of the future?

**DOI:** 10.1111/aos.14954

**Published:** 2021-07-16

**Authors:** Karen Frudd, Sobha Sivaprasad, Rajiv Raman, Subramanian Krishnakumar, Yeddula Rebecca Revathy, Patric Turowski

**Affiliations:** ^1^ Institute of Ophthalmology University College London London UK; ^2^ NIHR Moorfields Biomedical Research Centre Moorfields Eye Hospital London UK; ^3^ Vision Research Foundation Sankara Nethralaya Chennai Tamil Nadu India

**Keywords:** biomarker, diabetic retinopathy, serum, plasma, point‐of‐care testing

## Abstract

With the increasing prevalence of diabetes in developing and developed countries, the socio‐economic burden of diabetic retinopathy (DR), the leading complication of diabetes, is growing. Diabetic retinopathy (DR) is currently one of the leading causes of blindness in working‐age adults worldwide. Robust methodologies exist to detect and monitor DR; however, these rely on specialist imaging techniques and qualified practitioners. This makes detecting and monitoring DR expensive and time‐consuming, which is particularly problematic in developing countries where many patients will be remote and have little contact with specialist medical centres. Diabetic retinopathy (DR) is largely asymptomatic until late in the pathology. Therefore, early identification and stratification of vision‐threatening DR (VTDR) is highly desirable and will ameliorate the global impact of this disease. A simple, reliable and more cost‐effective test would greatly assist in decreasing the burden of DR around the world. Here, we evaluate and review data on circulating protein biomarkers, which have been verified in the context of DR. We also discuss the challenges and developments necessary to translate these promising data into clinically useful assays, to detect VTDR, and their potential integration into simple point‐of‐care testing devices.

## Introduction

Diabetes mellitus (DM) is a complex group of diseases characterized by high blood glucose levels due to either an inability to produce insulin or an insensitivity to insulin. The number of adults with any form of diabetes worldwide is estimated to have quadrupled from 108 million in 1980 to 463 million in 2019 (International Diabetes Federation, 2019). This equates to a doubling of % incidence across the population (Zhou et al., 2016). Hyperglycaemia, caused by diabetes, is a major risk factor for microvascular complications of diabetes. Diabetic retinopathy (DR) is a highly prevalent complication of diabetes throughout the world, with around 35% of people with diabetes thought to also have DR. The worst outcome of DR is blindness; 10% of people with diabetes have VTDR, and, as such, DR is a leading cause of acquired blindness in the adult population (Cheung et al., 2010; Yau et al., 2012).

Diabetes is a global disease and has not spared any nation. Many low‐ and middle‐income countries (LMICs) such as India and high‐income countries such as China are now facing a high public health burden due to diabetes (in China, the prevalence of diabetes rose from 0.67% to 9.7% between 1980 and 2008 (Yang et al., 2010)) and consequently increasing levels of DR. Whilst early detection and tight control of risk factors have decreased DR prevalence in some western countries (Wong et al., 2009; Liew et al., 2014; Liew et al., 2017; Claessen et al., 2018), this is not the case in LMICs where all forms of DR continue to be on the rise (Leasher et al., 2016; Flaxman et al., 2017). As the disease is largely asymptomatic in its early stages and thus not detected by ophthalmic examination, there are no recommended treatments other than control of risk factors, until more advanced pathology is identified. Diabetic retinopathy (DR) is currently diagnosed through imaging of the retina, revealing changes consequent to damage of the retinal vasculature. This requires specialist equipment and trained practitioners to both operate cameras and grade the images and is highly effective and efficient to detect referable cases of DR, as documented in countries with comprehensive healthcare systems. However, the ever‐increasing number of patients with diabetes precludes the sustainable use of retinal imaging for universal and routine screening (Vujosevic et al., 2020). For many countries, the cost of DR screening and treatments runs into the tens of millions and likely into the hundreds of millions when taking into account absence from work (Aspelund et al., 2011; Andersson et al., 2020). In the case of many LMICs, health care provision is disparate and often privately funded, and so costly routine screening for DR by current examination pathways is simply not a viable option. Simpler and more cost‐effective tests for DR are actively sought as they would benefit both high‐ and low‐income countries. Indeed, new risk stratification pathways and novel technologies, including more portable imaging equipment, the automation of grading and the data integration into telemedicine pathways are currently trialled and validated (Natarajan et al., 2019; Karakaya & Hacisoftaoglu, 2020). Emerging technologies and cost assessments for new retinal screening modalities have recently been extensively discussed elsewhere (Vujosevic et al., 2020). Here, we will instead focus on reviewing promising blood‐based protein markers that have potential to detect DR. Whilst many have withstood solid verification studies, caution is advised. Many markers have shown promise and specificity in detecting DR but may also be linked to the diabetic state generally, to inflammation or to parallel vascular morbidity. Thus, only large‐scale clinical validation will reveal if simple and cheap blood tests, accessible to all people with diabetes, can be considered as an effective option in the arsenal of DR screening pathways.

## Clinical features and current methods for diagnosis and monitoring of DR

DR is a progressive disease, which visibly affects the retinal vasculature. The initial instability of the vasculature eventually leads to microaneurysms and haemorrhages, and the consequent hypoxia triggers neovascularisation and, with breakdown of the delicate neuroretinal homeostasis, loss of visual acuity. Diabetic retinopathy (DR) is classified by observable clinical features of varying severities. In the early stages, the disease is asymptomatic and monitored for worsening but not treated. It is only in the later stages where there is a risk of vision loss that therapeutic intervention is applicable. It is widely recognized that effective screening and prompt intervention at the stage of VTDR limit losses in visual acuity (Jampol et al., 2020; Mansour et al., 2020). Indeed, clinical trials demonstrate that timely treatment for DR can reduce the risk of severe visual loss (ETDRS, 1991) (Wells et al., 2016).

The early stages of DR are referred to as non‐proliferative diabetic retinopathy (NPDR) and are characterized by the emergence of initial damage to the retinal vasculature. Observable microaneurysms are usually the first sign of NPDR, and the disease is classified as mild if these are the only retinal lesions observed. Individuals with moderate NPDR have more microaneurysms and may also have evidence of intraretinal haemorrhage, venous beading and other microvascular abnormalities. If large numbers of these abnormalities are present throughout the retina but there is no evidence of neovascularisation, then severe NPDR will be diagnosed. Once evidence of neovascularisation is seen, proliferative DR (PDR) is diagnosed, which can lead to loss of vision and will require intervention (Wilkinson et al., 2003; Core NDESP team, 2012). A further complication from DR is the development of diabetic macular oedema (DMO). DMO is characterized by the presence of hard exudates, thought to be leaked lipids which appear as yellow or whitish deposits with either sharp or diffuse margins in fundus images. It is also accompanied by thickening of the retina, generally revealed by optical coherence tomography (OCT). DMO is categorized as mild, moderate or severe depending on the extent of hallmark alterations of the retina. Typically, if these are located at the centre of the macular, the disease is severe and results in moderate visual loss if left untreated.

DR is currently effectively diagnosed and graded by imaging (Goh et al., 2016). Colour fundus photography of the retina in seven overlapping fields has been the gold standard for almost 30 years; however, this type of test is time‐consuming and can result in reduced patient compliance (ETDRS, 1991; Williams et al., 2004). In practice, up to three fields of fundus photography can provide adequate diagnostic power (Aptel et al., 2008; Vujosevic et al., 2009). One of the main downsides of colour fundus imaging is the difficulty in detecting DMO in 2D images. Optical coherence tomography (OCT), on the other hand, allows 2D and 3D analysis of the retina, showing changes in retinal architecture and thickness (Drexler & Fujimoto, 2008). Overall, this form of diagnosis is most accurate but also more costly. Not all people with diabetes, particularly in LMICs, can be subjected to annual retinal photographic screening due to the complexity and cost of this screening pathway and need for trained human resources (Vujosevic et al., 2020). To alleviate this problem, hand‐held cameras for fundus photography are being trialled, but it will still not enable universal coverage and frequent systematic retinal evaluation. Therefore, there is an unmet need to identify those at risk of blindness from the population with diabetes so that they can be triaged to confirmatory retinal screening test.

In most countries, where this screening is used, people with diabetes are divided into risk categories, which forms the basis for the regularity of their check‐ups, which again relies on retinal imaging. As only 8 to 10% of people with diabetes ever develop VTDR, isolating this group early will save much time and money. The availability of large datasets on individuals with DR has allowed some groups to devise algorithms, stratifying the risk of disease progression. Such methods, based on factors including duration of diabetes, HbA1c, systolic blood pressure, gender, and retinopathy grade, allow for more flexible screening intervals for those at lower risk and thus can reduce annual costs significantly (Aspelund et al., 2011; Broadbent et al., 2021). Whilst potentially highly effective for countries with defined DR treatment pathways, this type of monitoring is currently impractical and unachievable for LMICs where many of these factors are not routinely measured (Sivaprasad et al., 2020). Indeed, one study from India found that many individuals only seek help once their vision has begun to deteriorate (Shukla et al., 2016).

More recently, it has been recognized that DR also involves retinal neurodegeneration, which can develop in the absence of clinically diagnosed microvascular disease (Sohn et al., 2016; Simó et al., 2018). Diabetic retinal neuropathy structurally affects a wide variety of non‐vascular retinal cells and leads to measurable functional deficits (e.g. by electroretinogram). Whilst an extension of the currently used classification schemes such as the ETDRS has been proposed to incorporate novel technological advances and insights into DR pathogenesis including the comprehensive use of multimodal biomarkers (Abramoff et al., 2018), it should be noted that non‐invasive technologies to detect neuropathy are even more resource‐consuming than the imaging described above. Thus, additional focus on circulating biomarker may be of significant value to detect diabetic retinal neuropathy cost‐effectively. Indeed, and in analogy to cerebral neurodegeneration (Ashton et al., 2020), the degenerating retina may give rise to tractable biochemical and molecular changes in the circulation, albeit possibly to a smaller extent due to its much smaller size.

Taken together, a unique blood profile that identifies individuals, who will develop VTDR, would revolutionize DR screening in all countries.

## Pathogenesis of DR

Hyperglycaemia triggers multiple biochemical reactions, which contribute to the development and pathogenesis of DR (Brownlee, 2001). Oxidative stress, inflammation, accumulation of advanced glycation end products (AGEs), activation of protein kinase C (PKC), and dysregulation of the polyol and renin–angiotensin pathways can all contribute to vascular endothelial dysfunction leading to increased vascular permeability and/or neovascularisation, with no single process predominating (Cheung et al., 2010; Pusparajah et al., 2016; Wu et al., 2018; Antonetti et al., 2021).

Increased levels of circulating glucose during hyperglycaemia lead to surges in non‐enzymatic glycosylation of proteins such as haemoglobin and basement membrane proteins. During persistent hyperglycaemia, as in DM, this initially reversible glycosylation becomes irreversible and leads to the formation of AGEs (Brownlee et al., 1988; Stitt, 2010; Xu et al., 2018). Accumulation of AGEs in the retina induces pericyte apoptosis, increased production of endothelial growth factors and subsequent neovascularisation, and increased inflammation, all prevalent hallmarks of DR. Increased flux through the hexosamine (fructose‐6‐phosphate to UDP‐GlcNAc) pathway also leads to increased modification of proteins by o‐linked glycosylation, further exacerbating a hyperglycaemic state (Brownlee, 2001).

High glucose concentrations also dysregulate glucose metabolism and in particular the polyol pathway, which converts glucose to sorbitol and then fructose (Safi et al., 2014). Enzymes of this pathway utilize both NADPH and NAD+, and during glucose‐induced overload, large amounts of fructose will be produced at the expense of NADPH (Gabbay, 1973). This, in turn, results in an increased ratio of oxidized to reduced glutathione, and oxidative stress (Lorenzi, 2007).

Hyperglycaemia, through an excess of glycolytic intermediates, also leads to *de novo* synthesis of diacylglycerol (DAG), an activator of protein kinase C (PKC) (Koya & King, 1998; Guzik et al., 2002). In cultured endothelial cells, PKC activation causes permeability (Lynch et al., 1990). PKC activation also reduces endothelial vasodilation by dysregulation of endothelial nitric oxide synthase (eNOS) and upregulation of vasoconstrictors. In non‐endothelial vascular cells such as smooth muscle cells and pericytes, PKC activation causes further vascular dysregulation. Due to the wide range of detrimental effects from PKC activation during DR, many studies have tested inhibitors for different isoforms of PKC *in vitro* and *in vivo* with mixed results (Davis et al., 2009; Geraldes & King, 2010; Wu et al., 2018).

Many of these hyperglycaemia‐induced alterations of the vasculature and underlying neuronal‐glia networks also result in non‐specific inflammatory and oxidative stress responses, with increases in inflammatory mediators, such as IL‐1β, IL‐6, IL‐8 and MCP‐1 reported in plasma, serum and the vitreous and aqueous humour of DR patients (Youngblood et al., 2019). Naturally, DR shares many pathogenic mechanisms with DM, but also diabetic nephropathy (DN), another microvascular complication of DM. In both DR and DN, vessel stability and integrity are compromised, resulting in loss of function of the eye and kidney, respectively. Importantly, both of these microvascular complications of DM are risk factors for each other.

Persistent hyperglycaemia is considered a strong risk factor for the progression of DR. The Diabetes Control and Complications Trial (DCCT) reports that aggressive glycaemic control, along with control of blood pressure and circulating lipids, reduces DR progression in those with type‐1 DM (Hainsworth et al., 2019). In a recent data‐driven environment‐wide association study, HbA1c has also been recognized as the strongest risk factor among over 400 laboratory parameters (Blighe et al., 2020) (see also below).

## Biomarkers as tools for clinical assessment

A blood‐based biomarker test for DR could provide a rapid, cost‐effective and patient‐friendly means of screening at the population level to identify those at risk of VTDR, broadening access to care globally. Biomarkers can identify disease and even subclinical disease, but are also used to monitor clinical response to treatments (Lyons & Basu, 2012). Therefore, biomarkers can be diagnostic, prognostic and predictive, and their purpose needs to be defined early. The best biomarkers are specific and easily monitored by non‐invasive or minimally invasive methods, such as a blood test. For DR, many studies have focused on components in ocular fluids (Ma et al., 1996; Garcia‐Ramirez et al., 2007; Kim et al., 2007; Gao et al., 2008; Simo et al., 2008; McAuley et al., 2014). As delicate surgical procedures are required to obtain them, they are clearly not practical for high‐throughput screens at a population level. Nevertheless, many such studies have led to important insight into the pathogenesis of DR, e.g. the involvement of the kallikrein–kinin system (Liu & Feener, 2013), or formed the basis for subset selection in blood‐based verification studies (Kim et al., 2007; Jin et al., 2016).

**Fig. 1:**
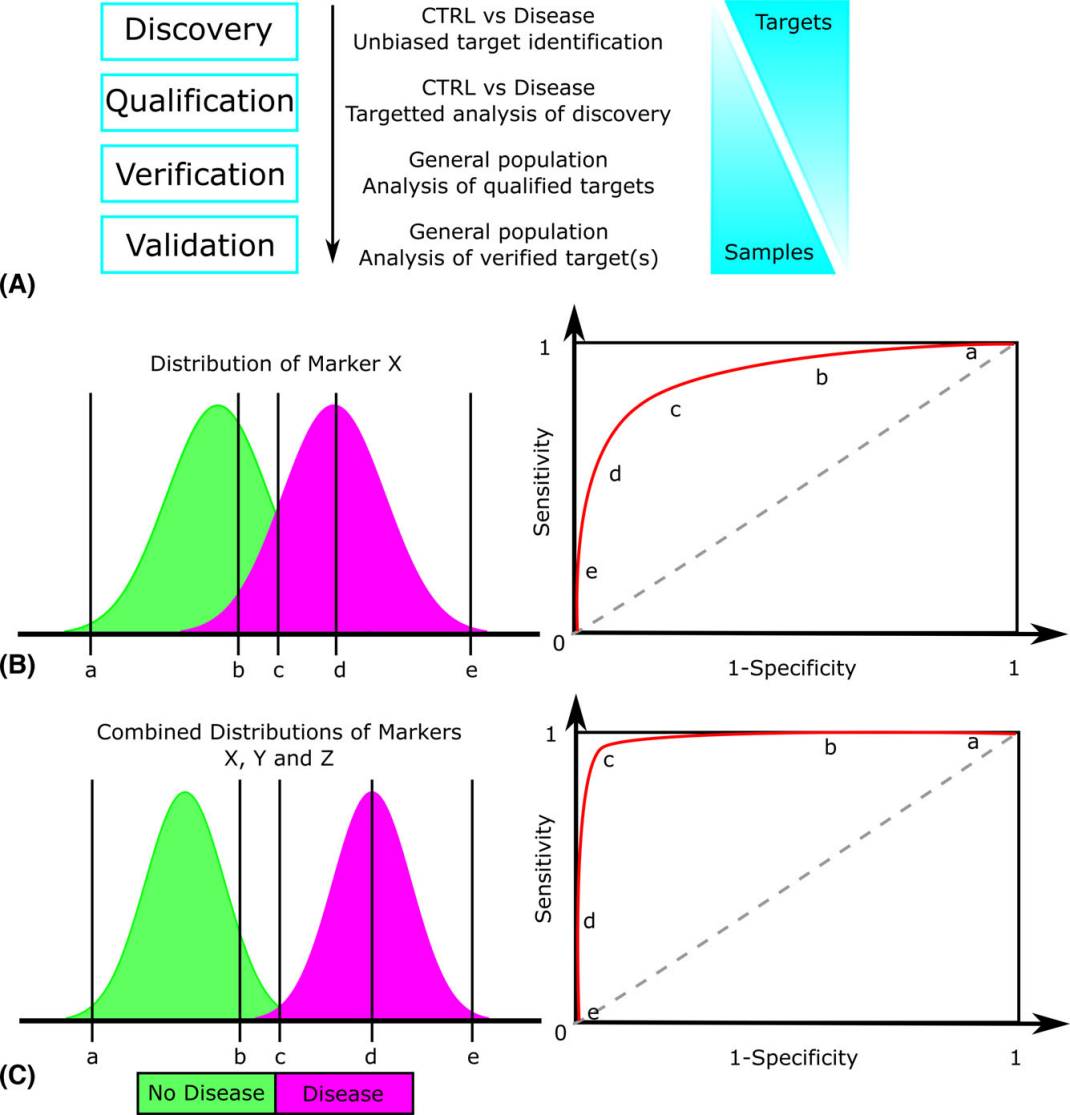
The stages of biomarker development. A, Typical stages of biomarker research indicating the types of sample required and the relative number of targets and patient samples used at each stage. B, Potential distribution of a marker (X) in disease and control. Points a–e demonstrate how different thresholds relate to the sensitivity and specificity of this marker using ROC curve analysis. C, The contribution of multiple markers could be used to improve a tests ability to distinguish between disease and no disease. In this case, data from multiple markers can be combined into one value by principle component analysis (or similar), which may then result in two distinct populations. This results in an AUC closer to 1 with increased sensitivity and specificity.

Typically, biomarker development needs to progress through several stages before a clinically useful end‐point is reached. These stages are often referred to differently but, broadly speaking, involve the following: a discovery step, whereby distinct control and target samples are tested in an unbiased way for any differences; a qualification step, where feasibility of identified markers is assessed in relation to the human disease of investigation; a verification step, where the specificity of markers is tested in a wider population‐based sample set; and finally if a marker has passed all of these stages, it will have to be validated in target patient groups using an optimized clinical assay (Fig. 1A). Discovery and qualification are usually focused on demonstrating sensitivity, whereas verification and validation are concerned with specificity (see also below). The required sample number will generally increase through biomarker development, while the number of targets assessed will decrease. Importantly, whilst verification of protein biomarkers is often still done using medium‐to‐high‐throughput methods such as MS, validation requires the development of a clinically robust (usually antibody‐based) assay for each marker under investigation. An overwhelming majority of preclinical biomarker candidates never make it to clinical use and some of those that do are ineffective due to failures in either the analysis or experimental design of the above stages. This may well be due to biomarker development being led by specialists of the disease rather than of biomarker development. Indeed, many specialized articles describe in detail all stages of biomarker development, highlighting associated pitfalls and the importance of consistency throughout the process (Rifai et al., 2006; Ioannidis & Bossuyt, 2017).

Importantly, biomarker data must be carefully computed to reveal their desired diagnostic, prognostic and predictive value. Distribution plots for disease and non‐disease groups reveal the overlap between the two. Theoretically, if all cases have reliably different values than all non‐cases – with no overlap – then a perfectly accurate prediction is possible. In practice, considerable overlap exists and models of discrimination are used to assess how well a given biomarker separates the target groups. In biomarker research, the discrimination is most often measured using receiver operating characteristic (ROC) curves, or *c* statistics, with the area under the curve (AUC) used to distinguish the discriminating power of different models. For ROC curves, sensitivity (the ability to detect true positives) is plotted against 1‐specificity (the ability to detect true negatives) across a range of thresholds creating a curve of increasing sensitivity with decreasing specificity (Fig. 1B, C). The area under the curve (AUC) gives a general measure of the accuracy of the test, with 1 indicating perfect prediction and 0.5 an equal likelihood of predicting disease or no disease, regardless of the biomarker value (Hoo et al., 2017). Points along the curve can be used to assess the relative specificity and sensitivity under those conditions and thus determine suitable inclusion or exclusion thresholds depending on the test and relative impact of misdiagnosis. Relying solely on *c* statistics and AUC has been criticized, in particular, when used for risk prediction (Cook, 2007). When the study cohort is representative of the general population, a large majority of cases will be non‐disease with similar measures, whereas the disease cases will be much fewer but with greater variation in measure. Thus, a biomarker with a clinically significant odds ratio may show little discriminative power by *c* statistics; many risk factors used for cardiovascular risk prediction today would not be considered on the basis of AUC discrimination. Additional tests such as likelihood ratios, and further stratification of the disease cases may be required to ascertain the contribution of the measured parameter to the severity of disease.

Generally, individual molecules are used as biomarkers. While single biomarkers are simple to test for and analyse, they can only provide limited diagnostic information. Currently, only a few biomolecules have the required sensitivity and specificity to be used as reliable markers. Individual molecules, particularly those related to inflammatory pathways, could also be indicative of more than one disease, potentially leading to an incorrect diagnosis. A multi‐marker panel could provide a more detailed diagnosis for complex diseases such as DR (Blighe et al., 2020). Multiple risk factors and pathways impact the development and progression of DR; thus, selecting successful classifiers that work across the population is challenging. In particular, shared pathological mechanisms of DR with other diabetic complications such as DN, but also other eye diseases, such as age‐related macular degeneration, can be confounders. Multiple component biomarker panels have the potential for greater sensitivity, specificity and improved stratification of disease groups (Rusling et al., 2010).

Two verification studies from the Kim group suggest that the combination of multiple markers could enhance sensitivity and specificity of using blood‐borne proteins in the detecting DR (Kim et al., 2013; Jin et al., 2016). These two studies identify early‐stage DR biomarkers in plasma, through use of multi‐marker panels, to enable accurate identification of individuals with VTDR. Candidate DR markers were analysed by multiple reaction monitoring mass spectrometry in plasma samples from people with all stages of NPDR (mild, moderate and severe) and people with diabetes without retinopathy (No DR) as a control. Twenty‐eight and 15 candidate proteins, from each study, respectively, were found differentially expressed across the four disease groups. These markers were reduced by logistic regression analysis, in different combinations, to improve predictive power. Importantly, in each study, the combination of four markers stratifies mild NPDR and both mild and moderate NPDR cases, respectively, against No‐DR cases much better than any single protein marker in isolation. If such four‐marker panels withstood validation in a large clinical cohort, combined with inexpensive, high‐throughput techniques, a new method to screen for DR could be rapidly developed.

## Integrating biomarkers with sensors

Clinical biomarker testing traditionally requires samples to be taken by a medical professional, and then sent to a laboratory for analysis. Results can take several days, at which point the patient may need to visit their health care provider again to discuss the results and their options. Point‐of‐care testing (POCT) enables immediate readouts of results and, in some cases, enables patients to monitor their disease state themselves. Currently, this type of testing is used in a small number of instances, such as blood glucose monitoring and pregnancy tests. However, advancements in biomarker identification could make it possible to diagnose many more diseases at the point of care (POC). Biosensors can be employed as portable POC devices, designed to detect and quantify target biomarkers. They are powerful analytical tools in medical diagnostics and provide an attractive platform for monitoring highly prevalent diseases, such as diabetes and its complications. A biosensor can detect biological molecules such as proteins and nucleic acids or monitor antigen–antibody interaction, for example. To generate an easily interpretable readout, a biosensor requires an element that can detect the biomolecule of interest, which is connected to a suitable transducer, capable of converting the biochemical signal into a quantifiable readout (Sethi, 1994; Vo‐Dinh, 2008; Devi et al., 2020). POC biosensors can be used in the clinical setting or in the field to give instantaneous results, reducing the need for travel and delays in diagnosis. To be maximally effective, electrochemical biosensors need to be easy‐to‐use, minimally invasive, sensitive, and inexpensive. This will enable rapid identification of at‐risk patients with reduced dependence on centralized medical and laboratory facilities.

Integrated biosensors also often come with further practical benefits including low sample volume requirements, easy‐to‐use interfaces, portability and low energy requirements. These mean that, although some training may be required, highly skilled operators may not be necessary, and in some cases, patients may self‐administer their tests. Overall, the lower costs and portability associated with this style of testing are ideal for LMICs where patients are likely to be spread across remote areas.

As sample processing is minimal or completely absent, POC biosensors must have high sensitivity and specificity for their target analyte in their target sample type (blood, urine or tear fluid, for example). Furthermore, as discussed above, complex diseases, such as DR, may require a combination of markers for accurate diagnosis. Thus, multiplex detection of biomarkers is necessary to ensure precise diagnostics and reduce costs in comparison with performing multiple single tests. Optimisation will, therefore, be required to enable the measurement of differentially expressed biomarkers in complex biological fluids, without interference from other highly abundant proteins.

The biosensor field continues to grow at pace, and the focus is not only on new target molecules but also new materials with enhanced capabilities in detection and signal transduction (Dinesh et al., 2019). Novel techniques, which do not rely on antibody‐based detection, may also be developed (Devi et al., 2020; Shalini Devi et al., 2020). There is significant interest in developing single‐step detection of pathogenic RNA and DNA in blood samples, which could be extended to circulating non‐coding RNA. Rapid detection of targets in un‐processed sample fluids is a major problem in this field and requires reactions to be both highly specific and highly sensitive to identify low‐abundance species in complex mixtures. Portability and compatibility with current technologies such as those in smart phones are also crucial to the success of novel biosensors (Banik et al., 2021). Miniaturisation of electronic transducers and microfluidics technology will hopefully maximize portability and allow multiplexing capacity in small devices. Ultimately, new diagnostic tests need to be an improvement on central laboratory testing that is the current norm. In some cases, cost per test may be increased, but this may result in improved management of chronic diseases, which will reduce overall health care costs. In the case of DR, even replacing a small percentage of imaging required for diagnosis would result in a huge saving in both time and money.

## Markers

As referred to previously, circulating biomarkers could be useful, not only for identifying VTDR but also for monitoring and stratifying patients based on their responses to treatments. Other reviews have covered promising biomarkers for DR and cover different molecule types as well as sample source and potential quantification or qualification (Jenkins et al., 2015; Raffort et al., 2015; Pusparajah et al., 2016; Ting et al., 2016; Nath et al., 2017; Safi et al., 2018). Here, we only focus on the most advanced circulating, protein biomarker candidates, for which verification data is already available, and for which clinical validation appears feasible using existing antibody‐based platforms.

### Glycated Haemoglobin (HbA1c)

#### Biological Role

HbA1c is formed by the non‐enzymatic glycation of haemoglobin in the blood and, in healthy adults, accounts for 1–4% of total Hb (Rahbar, 2005). HbA1c reflects the average blood glucose concentration over the preceding 120 days due to the irreversible nature of the glycosylation and the circulating life of erythrocytes. It has been adopted as a measure for the presence of diabetes, alongside other blood glucose measurements (Goldstein et al., 2004). The long‐lived nature of Hb glycosylation means such modifications can progress to AGEs which, as discussed above, can contribute to the pathogenesis of diabetic complications, including DR. Chronic hyperglycaemia, associated with diabetes, is thought to be the key driver of pathological changes indicative of DR and is measured by elevated HbA1c values (Cheung et al., 2010). The EURODIAB prospective complications study found that HbA1c level was significantly correlated with inflammatory markers measured in diabetic individuals, suggesting a link between persistent hyperglycaemia and endothelial inflammation (Schram et al., 2003).

#### Evidence

Many studies have identified the link between elevated HbA1c and increased risk of developing DR and several focus on maintaining strict control of HbA1c to mitigate this risk. For example, the DCCT study demonstrated that intensive control of HbA1c (<6.05%) in insulin‐dependent diabetic patients reduced the incidence of DR significantly compared to less stringent control (DCCT, 1995). However, in this study, HbA1c and diabetes duration only explained ~10% of the difference in retinopathy risk, suggesting a significant contribution of other factors (Hirsch & Brownlee, 2010). More recently, Lind et al. reported that such strict control of HbA1c (<6.5%) does not confer a significant reduction in DR risk and increases the risk of hypoglycaemia in type‐1 diabetics. They identify a range of 6.5–6.9% as ideal to prevent the development of serious complications. The risk of major and mild complications was significant for individuals with HbA1c >8.6% and >7.0%, respectively (Lind et al., 2019). Similarly, a 2012 meta‐analysis of global data on DR prevalence identified an increase in prevalence of any DR from 18% to 51.2% when comparing HbA1c of <7 and >9 (Yau et al., 2012).

Not all studies find that Hba1c levels are statistically predictive of DR progression or severity however, likely due to differences in patient cohort and study design. For example, the Veterans Affairs Diabetes Trial found no benefit to strict glycaemic control with regard to DR risk; however, this group was mainly male, had a mean age of 60 years and around 40% had already experienced an adverse cardiac event (Duckworth et al., 2009).

More recently, Hba1c variability rather than its static value has been posed as a better predictor of DR risk. High variability between HbA1c measurements on successive clinic visits is predictive of new‐onset DR but not directly predictive of progression to worsening forms of DR. Interestingly, abrupt decreases in HbA1c, as well as increases, contribute to this (Kilpatrick et al., 2008; Kim et al., 2021). Furthermore, in a Cox regression model predicting DR risk, the addition of Hba1c variability improved predictive power (Hermann et al., 2014). It is important to note that HbA1c variability can be calculated in different ways and not all give the same results with respect to DR prediction (Foo et al., 2017). HbA1c is inextricably linked to diabetes and the risk of developing further complications, although it is clearly not the only factor or marker. Taken together, HbA1c levels are a good predictor of DR risk and could become a useful clinical marker, especially when combined with other clinical readouts (Blighe et al., 2020).

### Enzyme inhibitors

#### Alpha‐2‐macroglobulin (A2MG)

##### Biological Role

Alpha‐2‐macroglobulin (A2MG) is a major blood glycoprotein that functions as a proteinase inhibitor by physically entrapping a broad range of proteases including trypsin, thrombin and collagenase and delivering them to an endocytotic clearance pathway (Idiris et al., 2003; Wang et al., 2011). A2MG has also been implicated in immunomodulation and extracellular proteostasis (Borth, 1992; Armstrong et al., 1999; French et al., 2008). Alpha‐2‐macroglobulin (A2MG) is known to bind to growth factors and cytokines, including transforming growth factor‐β, tumour necrosis factor‐α (TNFα), interleukin 1β, interleukin 8 and vascular endothelial growth factor (LaMarre et al., 1991; Feige et al., 1996). Binding can result in degradation of the complex or stabilize circulating factors, depending on the form of A2MG, modulating immune and inflammatory responses.

##### Evidence

There has been a long‐standing link between A2MG levels and DM, and a correlation between levels of A2MG and HbA1c has been noted (James et al., 1980; Turecký et al., 1999). Characterisation of the salivary proteome in individuals with type 2 diabetes mellitus (T2DM) indicated that A2MG was incrementally increased in the saliva of those in a prediabetic state and further increased in those with diagnosed T2DM (Rao et al., 2009). It has long been known that circulating A2MG is elevated in people with diabetes compared to healthy controls and even that elevated A2MG is associated with the presence of DR (James et al., 1980; Gray et al., 1982; Takada et al., 2013; Yoshino et al., 2019). Indeed, A2MG has been identified as a marker for DR in several sample types including saliva (Rao et al., 2009), vitreous (Kim et al., 2007) and, importantly for this review, in plasma (Kim et al., 2013). The latter work shows that A2MG is increased in plasma of patients with mild NPDR compared to no DR and is useful, in combination with other markers, to identify patients with mild NPDR.

#### Cystatin C

##### Biological Role

Cystatin C (CysC) belongs to the evolutionarily well‐conserved cystatin type 2 superfamily of cysteine protease inhibitors (Barrett, 1986). Originally identified in cerebrospinal fluid in humans, CysC has since been identified in all mammalian body fluids and tissues where it regulates endogenous proteinases including multiple cathepsins and papain (Bobek & Levine, 1992; Turk et al., 2008). CysC is particularly abundant in brain tissue (Hakansson et al., 1996) where it is expressed by neurons, astrocytes, endothelial and microglial cells (Yasuhara et al., 1993; Palm et al., 1995; Miyake et al., 1996). Cystatin C (CysC) is also used as a marker of glomerular filtration rate as it is completely removed from the circulation in the kidney and then almost fully resorbed by proximal tubular cells for degradation. Circulating levels of CysC remain fairly constant, and some studies refer to it as a housekeeping protein; however, changes in expression have been associated with diseases such as cancer, neurodegenerative disorders, DN and cardiovascular disease (Mussap & Plebani, 2004; Jeon et al., 2011; Kim et al., 2018).

##### Evidence

Recent studies have reported a positive correlation between serum CysC levels and DR in T2DM patients (He et al., 2013; Wong et al., 2015; Kim et al., 2018). Importantly, He et al., (2013) observed that circulating CysC levels are linked to the severity of DR and could be a predictor of VTDR. The authors showed that, along with the duration of DM and HbA1c, CysC is a risk factor for DR. The risk of VTDR was increased 11‐fold in patients with serum cystatin C levels over 1.25 mg/L (He et al., 2013; Wong et al., 2015) and revealed that serum CysC in T2DM patients correlated positively with moderate DR, suggesting that CysC may play a role in the pathogenesis of DR, although the mechanisms are unclear. In the eye, CysC is secreted by the retinal pigment epithelium and could contribute to the progression of macular degeneration (Zurdel et al., 2002; Paraoan et al., 2010), thus explaining the correlation between serum CysC and DR. Notably, CysC has been analysed in serum using an electrochemical immunosensor with high sensitivity (Devi & Krishnan, 2020). Using 1.2 mg/L as a cut‐off value the device showed 85% accuracy in predicting DR in a small (*n* = 10) cohort of clinical samples.

### Plasma protein transport regulators

#### Apolipoproteins

##### Biological Role

Apolipoproteins are lipid‐binding proteins, which help to transport triglycerides, phospholipids and cholesterol in blood, CSF and lymph. Apolipoproteins solubilize circulating lipids by forming lipoproteins, which are vehicles for the transport of lipids in the intra‐ and extravascular space. Apolipoproteins belong to several groups (ApoA–ApoH); individual forms are differentially expressed and associated with different types of circulating lipoprotein particle including chylomicrons, HDL, LDL and VLDL. Most apolipoproteins can move between lipoprotein particles as they are remodelled and circulate in the plasma, a feature that is referred to as exchangeable. ApoAI, the major protein component of HDL (constituting ~70%), is produced primarily in the liver and small intestine and is crucial to the regulation of cholesterol homeostasis. Furthermore, it possesses antioxidant, anti‐inflammatory and atheroprotective properties and is involved in the anti‐clotting process (Yui et al., 1988). ApoCIII is a component of very‐low‐density lipoprotein (VLDL), constituting ~40% of its protein mass, and HDL (Sundaram & Yao, 2012). It regulates the secretion and clearance of VLDL and inhibits the activities of several fat‐metabolising enzymes (Mendivil et al., 2010). ApoAIV is secreted from intestinal enterocytes and is mainly associated with chylomicrons and is potentially involved in their assembly (Green et al., 1980). ApoB is a non‐exchangeable apolipoprotein, remaining with the same lipoprotein from synthesis to cellular uptake and degradation. ApoB is mainly associated with VLDL, with one of its forms constituting the ligand for the LDL receptor (Boren et al., 1998). ApoB is strongly associated with the risk of developing coronary artery disease.

##### Evidence

Elevated serum levels of ApoAI and ApoCIII are associated with T2DM risk (Onat et al., 2009; Brahimaj et al., 2017), and analyses of vitreous fluid demonstrate a positive correlation between ApoA1 levels and PDR (Simo et al., 2008). By contrast, circulating ApoAI levels are inversely associated with DR, according to severity, in several studies of people with diabetes (Sasongko et al., 2011; Hu et al., 2012; Moosaie et al., 2020). A recent study by Zhang et al., (2018) further confirmed the association between circulating ApoAI and risk of DR but also found a positive relationship for ApoCIII levels. Elevated ApoA1 levels could be a protective factor against DR, where a baseline serum level of ApoAI ≥ 7.4 μmol/L was associated with a decreased risk of DR. In contrast, baseline levels of ApoCIII ≥ 6.3 μmol/L and ApoCIII‐to‐ApoAI ratio ≥0.9 correlated with an increased risk of DR (Zhang et al., 2018). Further studies by Chung et al. and Moosaie et al. also report an inverse relationship between ApoA1 and DR severity and a positive correlation of ApoB/ApoAI ratio to DR severity (Chung et al., 2019; Moosaie et al., 2020). Patients with type‐1 diabetes in the DCCT/EDIC cohort with severe retinopathy had significantly higher circulating ApoCIII concentration, compared to those with moderate or mild retinopathy (Klein et al., 2005). Kim et al., (2013) describe strong correlations between apolipoproteins in plasma with differing severity of DR. ApoCIII, ApoAII and ApoAIV are reduced in mild and moderate NPDR, compared to controls without retinopathy. ApoAI and ApoC1 are elevated in mild NPDR, but ApoC1 only in moderate NPDR (Kim et al., 2013).

#### Afamin (AFM)

##### Biological Role

Afamin, an albumin superfamily member, shares 55% amino acid similarity with albumin. It is primarily expressed in the liver and secreted into the bloodstream (Lichenstein et al., 1994) and noted for its high degree of glycosylation (Lichenstein et al., 1994; Araki et al., 1998). It is highly abundant in plasma but can also be found in follicle, seminal and cerebrospinal fluid (Voegele et al., 2002). Its role in vitamin E‐binding has been reported by several groups, and its ability to transport vitamin E across the blood–brain barrier has implications for neuroprotection (Heiser et al., 2002; Jerkovic et al., 2005; Kratzer et al., 2009).

##### Evidence

Strong correlations exist between serum afamin and the development of metabolic syndrome and high BMI (Kronenberg et al., 2014). A population‐based study on T2DM, including more than 20 000 individuals, also showed increased afamin concentrations in individuals with T2DM (Kollerits et al., 2017). Proteomics analysis showed decreased afamin expression in plasma from DR patients compared to no DR (Lu et al., 2013). Kim et al., (2013) point to its usefulness as a marker for DR since afamin, in combination with some of the other target proteins mentioned above, improves specificity in distinguishing moderate NPDR from T2DM patients with no DR.

#### Retinol binding protein 4 (RBP4)

##### Biological role

Circulating RBP4 binds and transports retinol (vitamin A), taking it from the liver to its target peripheral tissues. Retinol binding protein 4 (RBP4) solubilizes retinol, limiting the free amount in the circulation, which would otherwise be toxic. Many studies have identified links between RBP4, retinol, retinoic acid and obesity and its related conditions (Graham et al., 2006). These interactions and their implications have been reviewed comprehensively (Zabetian‐Targhi et al., 2015; Olsen & Blomhoff, 2020).

##### Evidence

Several studies have reported positive correlations between circulating RBP4 levels and T2DM or DR. Takebayashi et al. found serum RBP4 to be elevated in patients with diabetes compared to healthy controls and to be correlated positively with other markers of T2DM. Furthermore, RBP4 is elevated in patients with PDR compared to DR and non‐DR (Takebayashi et al., 2007). A similar trend was seen in another study where a significant positive correlation was also reported between serum RBP4 and urine albumin excretion rate (Li et al., 2010). More recently, in a cohort of 287 T2DM patients and 150 healthy controls, Li et al., (2018) found RBP4 to be significantly elevated in T2DM patients with DR or VTDR; the AUC was found to be 0.79 and 0.9 for DR and VTDR, respectively. However, other studies have reported a decrease in serum RBP4 with DM or simply no difference in circulating RBP4 in patients with DR (Akbay et al., 2010; Zhang et al., 2019a, 2019b). Due to the apparent links between RBP4 and metabolic disorders, correcting for BMI, fat deposition and urine albumin excretion may be key to elucidating genuine links between RBP4 levels and disease status.

### Coagulation cascade mediators

#### Complement cascade proteins

##### Biological role

The complement cascade is a key component of the innate immune system, which modulates various immune and inflammatory responses (Walport, 2001a, 2001b). The complement system is always active at a basal level, but its activity is monitored by complement regulators. It is now recognized that chronic, low‐grade inflammation and innate immune system over‐activation are features and influencers of T2DM (McLaughlin et al., 2017; Saltiel & Olefsky, 2017). Recently, circulating exosomes have been postulated as potential activators of complement in diabetic models (Huang et al., 2018). Elevated, circulating complement factor B (CFB) increases the risk of endothelial dysfunction (Hertle et al., 2016), which may lead to coronary heart disease (Donahue et al., 2006). Complement factor B (CFB) binds component C3 forming C3B, contributing to the formation of the membrane attack complex (Ricklin et al., 2010). Therefore, CFB is essential for pathogen clearance and host cell apoptosis. CFH is a soluble serum glycoprotein that regulates the function of the alternative complement pathway in blood and on cellular surfaces.

##### Evidence

The complement system has been implicated in the pathogenesis of DR and related conditions. Increased circulating CFB has been found in south Asian populations at risk of developing T2DM (Somani et al., 2012), and expression of CFB in adipose tissue has a strong correlation with fasting glucose and circulating lipids (Moreno‐Navarrete et al., 2010). Several studies have identified increased expression of CFB in the vitreous of DR patients, which led to further investigation of these proteins as potential specific markers for DR (Garcia‐Ramirez et al., 2007; Gao et al., 2008). Wang et al. (2013a) showed that polymorphisms in CFH and CFB genes are associated with the development of DR and that the combined effect of CFH rs80029 and CFB rs1048709 results in a significantly increased risk of DR. Additional polymorphisms in the *CFH* and *CFB* genes are also correlated with a higher risk of developing age‐related macular degeneration (Gold et al., 2006; Liu et al., 2010), a disorder that shares many pathophysiological features with DR. Based on differential expression of complement proteins in the vitreous of DR patients (Kim et al., 2007), Kim et al., (2013) identified CFB, CFH and complement component C3 as potential circulating markers for DR. In this case, plasma levels were decreased in mild and moderate NPDR patients compared to non‐DR controls. An additional paper from the same group also identifies complement component C7 as a circulating marker for DR, with ROC curve analysis showing the highest AUC (0.85) of any single marker analysed (Jin et al., 2016). Upon analysis of vitreous and serum samples from PDR, NPDR and healthy controls, Shahulhameed et al. identified a decrease of CFB and an increase in CFH in the vitreous of PDR patients. In contrast, CFH levels were downregulated in serum of these patients (Shahulhameed et al., 2020). Therefore, CFB and CFH could be accurate markers of DR, but the sample type appears crucial.

#### Factor 2 (F2, Thrombin)

##### Biological role

Blood coagulation crucially prevents blood loss and thrombin, a serine protease, plays a central role in the coagulation cascade. In a first step, its inactive precursor, prothrombin is cleaved to form active thrombin (Jeon et al., 2011). Thrombin then cleaves and solubilizes fibrinogen into strands of fibrin, an important step in the formation of clots. It also plays a key role in platelet activation and the catalysis of other coagulation‐related reactions. Further to its role in clot formation, thrombin is also a potent activator of angiogenesis and permeability during inflammatory responses (Maragoudakis et al., 2002; Mullins et al., 2009; Rathnakumar et al., 2016). Most mice lacking expression of thrombin die *in utero* due to defects in yolk sac vasculature, while those that are born succumb to haemorrhage on the first postnatal day (Sun et al., 1998). Mutations in the prothrombin gene, F2, lead to various forms of thrombosis and dysprothrombinaemia (Girolami et al., 2018).

##### Evidence

Kim et al., (2013) point to the usefulness of F2 (in combination with other markers) to detect and stratify DR. Additionally, proteomic analysis, as well as targeted ELISA analysis, showed increased prothrombin in vitreous samples from individuals with PDR compared to individuals with no diabetes (Gao et al., 2008; Abu El‐Asrar et al., 2016). Thrombin–anti‐thrombin III complex (TAT) is a parameter of coagulation and could act as a proxy for thrombin levels. Plasma and vitreous TAT levels have been shown to be significantly higher in patients with retinopathy (Asakawa et al., 2000) and have been shown to positively correlate with the severity of DR (Dan‐Brezis et al. 2020; Fujiwara et al., 1998). Given the many associations and activation steps thrombin is involved in, care must be taken in the comparison of studies.

#### Kallistatin (SerpinA4)

##### Biological role

Kallistatin (SERPINA4) is a serine protease inhibitor. Kallistatin binds to and inhibits the activity of tissue kallikreins which cleave kininogens to generate bioactive, pro‐inflammatory kinins (Chao et al., 1990; Zhou et al., 1992). Bradykinin has been implicated in the pathogenesis of DMO and DR due to its pro‐inflammatory and permeability‐inducing effects (Liu & Feener, 2013). Kallistatin activity triggers multifactorial effects, including vasodilation and inhibition of oxidative stress, inflammation, fibrosis and apoptosis, primarily by increasing NO formation via eNOS levels (Chao et al., 1990; Chao et al., 2006; Shen et al., 2008; Shen et al., 2010; Yin et al., 2010; Li et al., 2014).

##### Evidence

Kallistatin levels have been shown to be significantly reduced in the vitreous fluids of patients with PDR and the retinas of streptozotocin‐induced diabetic rats (Ma et al., 1996; Hatcher et al., 1997). Furthermore, Liu et al. showed that overexpression of kallistatin in an *in vivo* model ameliorates diabetes‐induced retinal leukostasis and vascular leakage, by inhibiting diabetes‐induced Wnt/β‐catenin signalling pathway activation (Liu et al., 2013). Interestingly, Kim et al. (2013) showed a stepwise fold‐increase in plasma kallistatin between control and mild NPDR subjects and also between mild NPDR and moderate NPDR subjects (Kim et al., 2013). This is in accord with other studies showing circulating kallistatin to be elevated in patients with diabetic vascular complications compared to control and subjects with diabetes with no vascular complications (Jenkins et al., 2010; McBride et al., 2014; El‐Asrar et al., 2015). Kallistatin may, therefore, be a more generalized marker for diabetes but does appear to be further increased in patients with additional complications such as DR and thus be a valuable marker in combination with others.

### Inflammatory markers

#### Lipoprotein‐associated phospholipase A2 (Lp‐PLA2)

##### Biological role

Lipoprotein‐associated phospholipase A_2_ (Lp‐PLA_2_) is a circulating phospholipase that binds to LDL‐cholesterol (LDL‐c) and HDL in the plasma. As an A2‐type phospholipase, Lp‐PLA_2_ hydrolyses modified polyunsaturated fatty acids within oxidized low‐density lipoprotein (oxLDL) releasing lysophosphatidylcholine (LPC) and oxidized non‐esterified fatty acids, which can elicit a range of pro‐inflammatory and pro‐apoptotic effects (Silva et al., 2011). Elevated Lp‐PLA_2_ has been proposed as a predictive biomarker for several vascular diseases including stroke (Oei et al., 2005), atherosclerosis (Katan et al., 2014) and coronary heart disease (Thompson et al., 2010). Macrophages and other pro‐inflammatory cells are a primary source of Lp‐PLA_2_ in the systemic circulation (Stafforini et al., 1990), although many ot her cells including endothelial cells also express this enzyme (Doublier et al., 2007).

##### Evidence

Lp‐PLA_2_ activity releases pro‐inflammatory lipids, which have been implicated in endothelial damage leading to disruption of the inner blood‐retinal barrier, observed in DR and DME. In vitro and in vivo studies with Lp‐PLA_2_ antagonists, darapladib and SB435495 (GlaxoSmithKline), have shown favourable responses in rats (Canning et al., 2016) and pigs (Acharya et al., 2017), improving visual loss by reducing retinal vascular leakage. Crucially, Lp‐PLA2 inhibition has demonstrated efficacy as a treatment for DMO, improving visual loss and reducing retinal thickness (Staurenghi et al., 2015). Interestingly, Siddiqui et al. showed that, in an adult Caucasian population, increased serum Lp‐PLA_2_ activity is not only associated with increased risk of development of DR but also with a transition to more advanced forms of DR (Siddiqui et al., 2018).

#### Leucine‐rich alpha‐2‐glycoprotein (LRG1)

##### Biological role

Leucine‐rich alpha‐2‐glycoprotein (LRG1) is a highly conserved member of the leucine‐rich repeat family of proteins (Andersen et al., 2010), which is involved in cell adhesion (Kobe & Kajava, 2001), granulocytic differentiation (O’Donnell et al., 2002), cell migration (Saito et al., 2002), signalling (Li et al., 2007), cell survival and apoptosis (Ai et al., 2008; Weivoda et al., 2008). Leucine‐rich alpha‐2‐glycoprotein (LRG1) has already been identified as a marker for various chronic inflammatory diseases, including rheumatoid arthritis and asthma (Fujimoto et al., 2015; Honda et al., 2016). Additionally, it can act as a mitogen for endothelial cells in tumour neovascularisation and, importantly, retinal neovascularisation (Wang et al., 2013b; Zhang et al., 2016). Leucine‐rich alpha‐2‐glycoprotein (LRG1) modulates endothelial transforming growth factor‐β (TGF‐β) signalling to promote angiogenesis (Wang et al., 2013b).

##### Evidence

Leucine‐rich alpha‐2‐glycoprotein (LRG1) exclusively localizes with the vasculature of various human tissues including the eye. Interestingly, its expression increases in response to the murine model of oxygen‐induced ischemic retinopathy (Wang et al., 2013b), which mimics neovascularisation seen in PDR. Plasma and intravitreal LRG1 has been described to be significantly increased in PDR patients, suggesting that LRG1 levels increase with DR progression (Chen et al., 2019; Hase et al., 2017; Zhang et al., 2019). This increase appears to be particularly detectable in more severe DR such as PDR but modest increases in milder forms of the disease could contribute to early detection.

#### Interleukin‐6

##### Biological role

Interleukin 6 (IL‐6) is a pleiotropic pro‐inflammatory cytokine that is mainly secreted by monocytes (Navarro et al., 1989) and binds to its specific receptor (IL‐6R) on the surface of cells. Also, IL‐6 can bind to soluble IL‐6R and thus directly activate cells. Interleukin 6 (IL‐6) promotes B‐cell maturation and T‐cell differentiation, while at the same time synergizing with TNFα and IL‐1 to promote a systemic inflammatory response (Romano et al., 1997; Skelly et al., 2013). IL‐6 production is rapidly upregulated in response to infections and tissue injuries; however, this is transient. As such, it is a key contributor to host defence through the stimulation of acute‐phase responses, haematopoiesis and immune reactions. The expression of Il‐6 is tightly controlled both transcriptionally and post‐transcriptionally. However, dysregulation of these mechanisms can lead to continual synthesis, which affects the pathology of chronic inflammation.

##### Evidence

IL‐6 has been implicated in the pathogenesis of DR because it is elevated in the vitreous fluid and blood of patients with DR (Schram et al., 2005; Kaviarasan et al., 2015; Feng et al., 2018; Yao et al., 2019). In the EURODIAB prospective complications study, circulating IL‐6, in combination with C‐reactive protein and TNFα, was able to stratify T1DM patients with no retinopathy, NPDR and PDR (Schram et al., 2005). Interleukin 6 (IL‐6) may be a key early indicator of DR as higher circulating levels were detected in children with DR, who, crucially, will have diabetes and DR for much shorter times than adults (Zorena et al., 2007). Furthermore, IL‐6 concentration in serum also positively correlates to the severity of DMO (Shimizu et al., 2002).

#### TNFα

##### Biological role

Tumour necrosis factor‐α (TNFα) is a primary cytokine linked to many cellular processes. Crucially, it can promote the production of reactive oxygen species, promote leukostasis and induce blood‐retinal barrier breakdown (Woo et al., 2000; Derevjanik et al., 2002; Chandrasekharan et al., 2007; Bradley, 2008). Tumour necrosis factor‐α (TNFα) has two receptors, TNFR1 and TNFR2, through which it signals and regulates cellular functions including proliferation, survival, differentiation and apoptosis. Tumour necrosis factor‐α (TNFα) is produced and secreted by macrophages and plays a pivotal role in inducing the cytokine cascade in many inflammatory diseases and is therefore under investigation as a therapeutic target for several diseases.

##### Evidence

As mentioned above, TNFα, in combination with other inflammatory markers, is an indicator of DR in T1DM patients (Schram et al., 2005). Circulating TNFα levels have been associated with retinopathy in several studies on patients with both T1DM and T2DM. In children with T1DM, this correlation was found to be a predictor of NPDR and was completely absent in healthy non‐diabetics (Zorena et al., 2007). Elevated TNFα level was found to be associated with severe retinopathy in T1DM patients with kidney disease; however, at a 15‐year follow‐up, this correlation was no longer observed (Klein et al., 2009). In African‐Americans with T1DM, it has been reported that baseline circulating TNFα is a predictor of PDR incidence as well as DME (Roy et al., 2013). Additionally, the TNFα level in tears is highly correlated with DR severity (Costagliola et al., 2013). Notably, moving towards alternative screening methods, a nanoparticle‐based sensor has been described in a proof‐of‐concept study to detect TNFα in tear fluid (Chuang et al., 2018).

### Basement membrane and extracellular matrix turnover markers

#### Collagen IV

##### Biological role

Collagen IV is an essential component of the basement membrane. It forms a mesh‐like network, surrounding epithelial and endothelial cells, supporting cellular adhesion, migration and wound healing (Boudko et al., 2018). Due to its integral role in the basement membrane, collagen IV acts as a scaffold for many different binding partners. It is degraded by specialist proteases, releasing subdomains important for signalling. Increased urinary collagen IV is a biomarker for diabetic nephropathy and microangiopathy (Haiyashi et al., 1992; Lee et al., 1994; Yagame et al., 1997).

##### Evidence

Collagen IV concentration has been evaluated in the serum, urine and vitreous of patients with diabetes and its associated microvascular complications. Elevated collagen IV in each of these fluids is associated with retinopathy or other diabetic complications, such as nephropathy or microalbuminuria, in both adults and children (Haiyashi et al., 1992; Yagame et al., 1997; Nicoloff et al., 2001. Plasma collagen IV levels were identified as indicative of severity of DN and DR (Lee et al., 1994). Kotajima et al., 2001 also found that collagen IV was elevated in the serum and also vitreous fluid of patients with DR. In the vitreous, this increase also correlated with disease duration.

#### Matrix metalloproteinases (MMPs)

##### Biological role

Matrix metalloproteinases (MMPs) are zinc‐dependent endopeptidases, which degrade and remodel all types of extracellular matrix, apart from polyglycan. Humans have at least 23 MMPs, out of a total of 28 found in vertebrates, which can be broadly subdivided based on their target proteins. Their functions involve tissue remodelling, wound healing, bone remodelling and cell migration, and, as such, MMPs can play roles in cancer metastasis and invasiveness as well as other diseases. In retinopathy, MMPs can degrade junction proteins, increase vascular permeability, exacerbate inflammatory responses, initiate cell death and promote neovascularisation (Kowluru & Mishra, 2017). In addition to this primary role, MMPs are also able to influence cell signalling behaviours through activation or inhibition of cell surface receptors. To prevent erroneous degradation, MMPs are produced as inactive pro‐enzymes that need to be proteolytically activated. The activity of MMPs is closely regulated by different factors including tissue inhibitors of metalloproteinases (TIMPs), which serve as their endogenous inhibitors. The balance between MMPs and TIMPs is crucial to their function in tissue homeostasis.

##### Evidence

MMP14 was found at significantly higher levels in vitreous from patients with PDR compared to non‐diabetic controls as well as in the retinae of diabetic rats (Abu El‐Asrar et al., 2018). Additionally, MMP14 was also higher in patients with active neovascularisation compared to those with stable PDR. MMP1 has been found in the vitreous of patients with PDR (around 40% of patients) but is not present in those without DM. Furthermore, a correlation was also seen between those expressing MMP1 and those with the highest levels of VEGF (Kwon et al., 2016). Circulating MMP1, MMP7 and MMP9 have also been found to be elevated in patients with diabetes, alongside the MMP/TIMP1 ratio, and these circulating factors are further increased in patients with DR (Jacqueminet et al., 2006) (Maxwell et al., 2001) (Abu El‐Asrar et al., 2014). In the EURODIAB study MMP2, MMP3, MMP10 and TIMP1 were higher in PDR patients with adjustment for age, sex, duration of DM and HbA1c; however, when these results were further corrected for CVD and albuminuria, only the MMP2 changes remained significant (Peeters et al., 2015). As CVD is a common complication of DM, these MMPs alone are unlikely to be suitable markers for DR or PDR.

### Other circulating factors

#### Advanced glycation end products

##### Biological role

As described above, AGE formation in response to hyperglycaemia is part of the DR pathogenesis (Brownlee et al., 1985). AGEs can perturb cellular function and also disrupt cell structure by accumulating in the vessel wall. In addition, they also act through specific receptors (RAGE) on endothelial cells, Muller glia, pericytes and retinal pigment epithelial cells by which they contribute further to vascular complications of diabetes. AGEs disrupt cellular homeostasis by modifying the extracellular matrix (ECM) but also by impacting on the action of hormones, cytokines and free radicals and the function of intracellular proteins (Brownlee et al., 1988).

##### Evidence

Two AGEs, in particular, have been proposed as biomarkers for DR. N‐Epsilon‐carboxymethyl lysine (N‐*ɛ*‐CML) is the most common circulating AGE and has been found at elevated levels in the serum of patients with diabetes and to an even higher extent in those with DR and other microvascular complications (Wautier et al., 2003; Boehm et al., 2004; Hirata & Kubo, 2004). Choudhuri et al., (2013) found that subjects with both PDR and NPDR had significantly increased total serum AGEs compared to no DR; however, the NPDR group had significantly higher levels of N‐*ɛ*‐CML than the PDR group. Kerkeni et al., (2012) showed that serum levels of AGEs were elevated in DR patients and also reported an increase in pentosidine, another AGE related to DR, in DR patients compared to controls. However, this may not be a highly specific marker for DR as, in the EURODIAB study, the association between pentosidine and DR was attributed to the duration of diabetes (Schram et al., 2005). To further complicate the presence of pentosidine in DR, Salman et al., (2009) found elevated levels in the early and moderate stages of NPDR, but this was lost once patients had developed PDR, as seen by some groups with N‐*ɛ*‐CML. Kidney disease is often a further complicating factor in the pathogenesis of DM. This may be key to levels of circulating AGEs in DM patients as AGE levels tend to increase with loss in kidney function (Hirata & Kubo, 2004).

#### Vascular endothelial growth factor (VEGF)

##### Biological role

Vascular endothelial growth factors (VEGFs) are a family of endothelial‐specific cytokines which have functions in both physiological and pathological angiogenesis of different vessel types throughout the body. VEGFA is the prototypical form, often just referred to as VEGF, responsible for endothelial homeostasis but also vascular permeability. Dysregulated levels of VEGF can lead to aberrant leakage and vessel growth and have been directly implicated in the pathogenesis of DR. VEGF also drives early events of DR pathogenesis by inducing ICAM‐1 expression, leading to leukocyte adhesion and blood‐retinal barrier breakdown (Joussen et al., 2002). Due to the roles of VEGFs in DR, anti‐VEGFs are increasingly used to treat advanced retinopathies; however, they are not effective in all patients (Ford et al., 2013). In this regard, it is interesting to note that, in at least one study, VEGF was not detectable in the ocular fluids of some patients with DR, which may explain why not all DR patients respond to anti‐VEGF treatment (Aiello et al., 1994).

##### Evidence

Many studies have described links between circulating VEGF levels and DR; however, these studies often do not agree on the degree of correlation or ability to predict disease severity. Increased serum VEGF levels have been linked to DR and raised HbA1c values (Celebiler Cavusoglu et al., 2007), which have also been associated with an increased risk of DM complications (Nordwall et al., 2015). Furthermore, several studies have shown that VEGF levels in serum are increased with the severity of DR (Celebiler Cavusoglu et al., 2007; Du et al., 2014). Levels of serum VEGF correlate positively with disruption of the external limiting membrane and the inner‐segment–outer‐segment junction, suggesting that increased serum VEGF is associated with severity of DR (Jain et al., 2013). Ozturk et al., (2009) reported a significant correlation between serum VEGF and severity of DR although there was no statistically significant difference between NPDR and PDR. A further study reported that although VEGF significantly increases in DM compared to controls, it is lower in PDR compared to NPDR (Suguro et al., 2008). Other studies, such as the one from Chaturvedi et al, found only a weak correlation between VEGF and the severity of DR, this time in plasma (Chaturvedi et al., 2001). A recent meta‐analysis of 29 different studies found that these showed overall that serum but not plasma VEGF levels were increased in DR patients compared to controls, with increases also correlating with severity of disease (Zhou et al., 2019).

## **Limi**tations of circulating protein biomarkers

Diseased tissues generally display molecular signatures related to their pathology and pathogenesis, and these can sometimes be utilized through tissue biopsies. However, serum and plasma are preferred for biomarker‐based tests: they can be considered a circulating representation of all body tissues, also reflecting disease‐specific molecular signatures. Discovery of proteomic signatures is often hampered due to the complexity and dynamic range of serum and plasma (often requiring predepletion of highly abundant constituents). In addition, with pathologies that are restricted to a relatively small proportion of the body, many specific biomarker changes cannot be detected reliably. This is undoubtedly an important factor for biomarkers of DR, as the retinal blood volume constitutes a small proportion of the total circulation. For instance, increased intraocular VEGF has been measured in the vitreous of all forms of DR, but changes in circulating levels do not reflect this robustly enough to justify its use as a blood‐based biomarker. In the case of pigment epithelium‐derived factor (PEDF), circulating levels are increased in patients with PDR, compared to those without, yet in ocular tissue, PEDF levels are lower in patients with late‐stage DR than in those without retinopathy (Jenkins et al., 2007; Li et al., 2012; McAuley et al., 2014). This may not be an issue, in theory, provided the results for circulating levels are consistent and reliable. However, it does pose questions regarding why this is the case and what altered levels of PEDF are indicative of. In this case, targeted basic science studies can show how biomarker levels correlate to pathogenesis (Elahy et al., 2014) and, in combination with more longitudinal studies on patients, could help to develop a more nuanced classification of disease. Lastly, as DR constitutes a complication of a complex systemic disorder, one should be wary of changes that may in fact not be specific for DR but possibly of generalized inflammation or vascular disease.

Biomarker validation is highly dependent on preanalytical specimen handling, which needs to be standardized to minimize technical variance between studies (also reviewed by (Rifai et al., 2006)). Several studies have demonstrated significant changes in the levels of analytes following different processing protocols. Additionally, protein biomarkers are not very robust analytes, which could make them disadvantageous in an outpatient setting, where the period between sampling and sample analysis can sometimes vary due to unforeseen delays. Other extreme conditions such as repeated freeze and thaw cycles can compromise protein stability in serum. Additionally, classical immunoassays, such as ELISA, are highly sensitive, but labour‐intensive and challenging to implement for multiplexing detection.

Biomarkers must be validated on large cohorts to determine usefulness across the general population. However, changes that are only significant in large cohorts may not provide sufficient specificity and sensitivity in individual patients. New biomarkers also need to be tested on diverse populations in case they have altered specificities based on gender, age, ethnicity or type of diabetes. Indeed, it has recently been suggested that people with diabetes can be more usefully subdivided into five groups, based on clinical characteristics, rather than the two more commonly used to date. These cohorts allow better stratification of disease outcomes and could provide an early indication of complications (Ahlqvist et al., 2018). None of the DR biomarker verification studies have correlated marker levels to these more advanced clinical subgroups of diabetes. In addition, DR classification often differs considerably between studies, thus making direct comparison difficult. In line with this, pre‐existing comorbidities, medications and other environmental factors could also alter biomarker levels and their relationship with DR. Consideration of such comorbidities is not always included in study design and analysis, which could explain, in part, some differences between reports. Nephropathy is a closely related microvascular complication to retinopathy and many studies describe a greater risk of retinopathy in patients with nephropathy and cardiovascular disease risk is also elevated in patients with existing diabetes complications (Hahr & Molitch, 2010; Son et al., 2011; Grunwald et al., 2012; Rajalakshmi et al., 2020). This is perhaps unsurprising as both diseases affect microvascular beds, dense with capillaries, and share many of the same risk factors including high HbA1c, duration of diabetes, hypertension and poor lipid control (Romero et al., 2007; Lee et al., 2014). Therefore, biomarkers may in fact stratify the high‐risk group of people with diabetes that should be triaged for at risk of complications. In addition, validation in multiple cohorts needs to be done before clinical pathways can be re‐designed to include biomarkers and biosensors.

## Future trends

Circulating biomarkers will continue to evolve with increased identification of markers, ongoing improvements in detection limits, and reduction of the operating cost and time. Furthermore, new technologies, including proteomics, metabolomics and genomics, will enable exploration of previously unavailable target molecules and will potentially lead to identification of novel biomarkers.

As discussed in this review, electrochemical biosensors have emerged as advantageous molecular sensing devices with the potential to benefit POC diagnostics (Shalini Devi et al., 2020). Furthermore, the emergence of nanotechnologies is providing new materials and methodologies for POC devices, reducing sample volumes and improving portability (Pirzada & Altintas, 2019). It is also becoming increasingly possible to couple devices to smartphones, allowing for at‐home testing and increasing the possibility to monitor complex conditions with regularity (Kou et al., 2020). Traditional tests for many conditions, such as diabetes, use antibody‐mediated detection to confirm the presence or quantity of a target analyte. Miniaturisation of this process must take into consideration the stability of the biological components, ease of sample preparation, as well as the cost and reliability of the device (Chen et al., 2020). Devices and reactions need to be particularly robust if patients are to self‐administer as there will be variations in compliance and environment.

The use of blood‐based biomarkers is ubiquitous throughout current medical practice and many types of molecule can be detected, including proteins, lipids and sugars. However, in recent decades the research community has been exploring additional metrics such as circulating RNAs and metabolic by‐products. There is a wealth of published data on the use of microRNA as biomarkers for DR as well as more novel omics approaches such as metabolomics (Raffort et al., 2015; Gong & Su, 2017; Zhang et al., 2017; Martinez & Peplow, 2019; Zhu et al., 2019); however, this is beyond the scope of this review. Additionally, proteomics techniques are being applied to different sample types to identify more specialized markers. As discussed in this review, and others, aqueous and vitreous humour are only obtained with invasive surgery and so not suitable for screening; however, tear fluid could be a non‐invasive sample source for detecting diseases of the eye (Csősz et al., 2017).

It is anticipated that any novel biomarkers will be embedded in current and future care and diagnostic pathways, and undoubtedly current screening methods and pathways will evolve at pace as well. Thus, for DR and many other retinal disorders, automated image analysis and cloud technologies are being harnessed to reduce the need for manual retinopathy grading (Trucco et al., 2013; Tufail et al., 2017). Recent work on the use of machine learning has shown that with a large amount of data, an algorithm can be trained to detect DR from fundus images (Takahashi et al., 2017; Ting et al., 2017). In addition, the use of smartphone cameras is being explored to improve the accessibility of imaging analyses. Progress is also being made in using non‐mydriatic cameras, thus further easing burden of intervention (Nderitu et al., 2021). Machine learning techniques promise to detect early changes in vasculature, which may be beyond the capabilities of any trained ophthalmologist, and thus will form a key part of future telemedicine. Nevertheless, they may continue to require high‐quality images, which cannot be easily obtained for the most remote patients, and data storage, processing and administration will continue be associated with considerable cost and requirement of expert input. Overall, this leaves a clearly defined role for a routine and cheap biomarker test, should it become available.

Whilst current biomarker development focuses on detecting and stratifying ongoing retinopathy, future studies should also explore if predictive molecular signatures can be identified. In addition, markers that predict the effectiveness of current interventions for individuals with DR could reduce costs and streamline clinical pathways considerably. For instance, even with aggressive anti‐VEGF treatment around 50% of patients have persistent macular oedema and moderate to no improvement in their visual acuity (Ford et al., 2013), suggesting a different treatment plan could have been more beneficial. However, developing predictive markers will require much more extensive longitudinal cohort studies fuelled by clearly defined preclinical candidates.

## Conclusion

Efficient, cost‐effective methods for monitoring DR and specifically for identifying early‐stage VTDR will be a game‐changer in the management of this disease, particularly in LMIC. Circulating biomarkers could be complementary to existing pathways, not only for identifying these patients but, also, for stratifying patients according to their treatment responses and monitoring their progress. Indeed, a more holistic approach to diagnosis and care of all microvascular complications of diabetes may be the most appropriate model, and circulating parameters are the best surrogate for such disease phenotypes. Effective collaboration between specialists would undoubtedly improve the risk stratification of individuals with diabetes. However, a cheaper screening marker may help stratify the population with diabetes better, so that the group at risk of complications can be triaged for more detailed screening of complications using gold standard tests. For example, DR is a costly disease in all countries, either through cost of treatment and monitoring or through the burden of blindness. Therefore, all available tools should be exploited to suit the means and requirements in each region or country.

At present, a selective marker for early‐stage DR remains elusive. In reality, it may be most achievable to identify people with diabetes most at risk of developing any form of microvascular complication and then further triage these people to the most appropriate specialists. For either of these outcomes, large, comprehensive studies are required comparing markers for different microvascular complications of DM.

If a blood‐based test or sensor can be developed, this could easily be incorporated into existing clinical settings or laboratories for onward referral to specialist care centres. Streamlining this diabetes care pathway will have significant immediate impact, especially in LMIC, where patients tend to self‐refer themselves when complications are already advanced and symptomatic. Nevertheless, the complexity of integrating a blood‐based test into some existing clinical practice should not be underestimated. Even regular HbA1c measurements are not accessible to many people with diabetes.

Many small studies have identified and verified potential circulating biomarkers for DR; however, none of these have been validated in large multi‐centre studies. Multiple potential confounders need to be addressed in the search for screening markers, including geographic, ethnic and genetic variations in the study populations as well as the varying phenotypes of DR. Therefore, large‐scale, collaborative, multi‐centre studies will be needed to conclusively validate and determine the reliability of the various biomarkers of DR.

## References

[aos14954-bib-0001] Abramoff MD , Fort PE , Han IC , Jayasundera KT , Sohn EH & Gardner TW (2018): Approach for a clinically useful comprehensive classification of vascular and neural aspects of diabetic retinal disease. Invest Ophthalmol Vis Sci 59: 519–527. 10.1167/iovs.17-21873. 29372250 PMC5786342

[aos14954-bib-0002] Abu El‐Asrar AM , Alam K , Nawaz MI et al. (2016): Upregulation of thrombin/matrix metalloproteinase‐1/protease‐activated receptor‐1 chain in proliferative diabetic retinopathy. Curr Eye Res 41: 1590–1600. 10.3109/02713683.2016.1141964. 27261371

[aos14954-bib-0003] Abu El‐Asrar AM , Mohammad G , Allegaert E et al. (2018): Matrix metalloproteinase‐14 is a biomarker of angiogenic activity in proliferative diabetic retinopathy. Mol Vis 24: 394–406. Retrieved from https://pubmed.ncbi.nlm.nih.gov/29853773, https://www.ncbi.nlm.nih.gov/pmc/articles/PMC5957543/. 29853773 PMC5957543

[aos14954-bib-0004] Abu El‐Asrar AM , Mohammad G , Nawaz MI et al. (2014): Relationship between vitreous levels of matrix metalloproteinases and vascular endothelial growth factor in proliferative diabetic retinopathy. PLoS One 8: e85857. 10.1371/journal.pone.0085857. PMC3877391 24392031

[aos14954-bib-0005] Acharya NK , Qi X , Goldwaser EL et al. (2017): Retinal pathology is associated with increased blood‐retina barrier permeability in a diabetic and hypercholesterolaemic pig model: Beneficial effects of the LpPLA(2) inhibitor Darapladib. Diab Vasc Dis Res 14: 200–213. 10.1177/1479164116683149. 28301218

[aos14954-bib-0006] Ahlqvist E , Storm P , Käräjämäki A et al. (2018): Novel subgroups of adult‐onset diabetes and their association with outcomes: a data‐driven cluster analysis of six variables. Lancet Diabetes Endocrinol 6: 361–369. 10.1016/S2213-8587(18)30051-2. 29503172

[aos14954-bib-0007] Ai J , Druhan LJ , Hunter MG , Loveland MJ & Avalos BR (2008): LRG‐accelerated differentiation defines unique G‐CSFR signaling pathways downstream of PU. 1 and C/EBPɛ that modulate neutrophil activation. J Leukocyte Biol 83: 1277–1285. 18272588 10.1189/jlb.1107751 PMC2376838

[aos14954-bib-0008] Aiello LP , Avery RL , Arrigg PG et al. (1994): Vascular endothelial growth factor in ocular fluid of patients with diabetic retinopathy and other retinal disorders. N Engl J Med 331: 1480–1487. 10.1056/nejm199412013312203. 7526212

[aos14954-bib-0009] Akbay E , Muslu N , Nayır E , Ozhan O & Kiykim A (2010): Serum retinol binding protein 4 level is related with renal functions in Type 2 diabetes. J Endocrinol Invest 33: 725–729. 10.1007/BF03346678. 20436266

[aos14954-bib-0010] Andersen JD , Boylan KL , Jemmerson R et al. (2010): Leucine‐rich alpha‐2‐glycoprotein‐1 is upregulated in sera and tumors of ovarian cancer patients. J Ovarian Res 3: 21. 20831812 10.1186/1757-2215-3-21 PMC2949730

[aos14954-bib-0011] Andersson E , Persson S , Hallén N , Ericsson Å , Thielke D , Lindgren P , Steen Carlsson K & Jendle J (2020): Costs of diabetes complications: hospital‐based care and absence from work for 392,200 people with type 2 diabetes and matched control participants in Sweden. Diabetologia 63: 2582–2594. 10.1007/s00125-020-05277-3. 32968866 PMC7641955

[aos14954-bib-0012] Antonetti DA , Silva PS & Stitt AW (2021): Current understanding of the molecular and cellular pathology of diabetic retinopathy. Nat Rev Endocrinol 17: 195–206. 10.1038/s41574-020-00451-4. 33469209 PMC9053333

[aos14954-bib-0013] Aptel F , Denis P , Rouberol F & Thivolet C (2008): Screening of diabetic retinopathy: effect of field number and mydriasis on sensitivity and specificity of digital fundus photography. Diabetes Metab 34: 290–293. 10.1016/j.diabet.2007.12.007. 18406188

[aos14954-bib-0014] Araki T , Haupt H , Hermentin P , Schwick HG , Kimura Y , Schmid K & Torikata T (1998): Preparation and partial structural characterization of alpha1T‐glycoprotein from normal human plasma. Arch Biochem Biophys 351: 250–256. 10.1006/abbi.1997.0564. 9514662

[aos14954-bib-0015] Armstrong PB , Quigley JPJD & Immunology C (1999): α2‐macroglobulin: an evolutionarily conserved arm of the innate immune system. Dev Comp Immunol 23(4–5): 375–390. 10426429 10.1016/s0145-305x(99)00018-x

[aos14954-bib-0016] Asakawa H , Tokunaga K & Kawakami F (2000): Elevation of fibrinogen and thrombin–antithrombin III complex levels of type 2 diabetes mellitus patients with retinopathy and nephropathy. J Diabetes Complications 14: 121–126. 10989319 10.1016/s1056-8727(00)00075-1

[aos14954-bib-0017] Ashton NJ , Hye A , Rajkumar AP et al. (2020): An update on blood‐based biomarkers for non‐Alzheimer neurodegenerative disorders. Nat Rev Neurol 16: 265–284. 10.1038/s41582-020-0348-0. 32322100

[aos14954-bib-0018] Aspelund T , Thornórisdóttir O , Olafsdottir E et al. (2011): Individual risk assessment and information technology to optimise screening frequency for diabetic retinopathy. Diabetologia 54: 2525–2532. 10.1007/s00125-011-2257-7. 21792613

[aos14954-bib-0019] Banik S , Melanthota SK , Arbaaz , Vaz JM , Kadambalithaya VM , Hussain I , Dutta S & Mazumder N (2021): Recent trends in smartphone‐based detection for biomedical applications: a review. Anal Bioanal Chem 413: 2389–2406. 10.1007/s00216-021-03184-z. 33586007 PMC7882471

[aos14954-bib-0020] Barrett AJ (1986): The cystatins: a diverse superfamily of cysteine peptidase inhibitors. Biomed Biochim Acta 45(11–12): 1363–1374. 3555466

[aos14954-bib-0021] Blighe K , Gurudas S , Lee Y & Sivaprasad S (2020): Diabetic retinopathy environment‐wide association study (EWAS) in NHANES 2005–2008. J Clin Med 9: 2005–2008. 10.3390/jcm9113643 33198349 PMC7696981

[aos14954-bib-0022] Bobek LA & Levine MJ (1992): Cystatins–inhibitors of cysteine proteinases. Crit Rev Oral Biol Med 3: 307–332. 1391414 10.1177/10454411920030040101

[aos14954-bib-0023] Boehm BO , Schilling S , Rosinger S , Lang GE , Lang GK , Kientsch‐Engel R & Stahl P (2004): Elevated serum levels of Nɛ‐carboxymethyl‐lysine, an advanced glycation end product, are associated with proliferative diabetic retinopathy and macular oedema. Diabetologia 47: 1376–1379. 10.1007/s00125-004-1455-y. 15258735

[aos14954-bib-0024] Boren J , Lee I , Zhu W , Arnold K , Taylor S & Innerarity TL (1998): Identification of the low density lipoprotein receptor‐binding site in apolipoprotein B100 and the modulation of its binding activity by the carboxyl terminus in familial defective apo‐B100. J Clin Invest 101: 1084–1093. 10.1172/jci1847. 9486979 PMC508660

[aos14954-bib-0025] Borth WJTFJ (1992): Alpha 2‐macroglobulin, a multifunctional binding protein with targeting characteristics. FASEB J 6: 3345–3353. 1281457 10.1096/fasebj.6.15.1281457

[aos14954-bib-0026] Boudko SP , Danylevych N , Hudson BG & Pedchenko VK (2018): Chapter 10 ‐ Basement membrane collagen IV: Isolation of functional domains. In: Mecham RP (ed.). Methods in Cell Biology, Vol. 143. San Diego, CA: Academic Press 171–185. 29310777 10.1016/bs.mcb.2017.08.010 PMC5828530

[aos14954-bib-0027] Bradley J (2008): TNF‐mediated inflammatory disease. J Pathol 214: 149–160. 10.1002/path.2287. 18161752

[aos14954-bib-0028] Brahimaj A , Ligthart S , Ikram MA , Hofman A , Franco OH , Sijbrands EJ , Kavousi M & Dehghan A (2017): Serum levels of apolipoproteins and incident type 2 diabetes: a prospective cohort study. Diabetes Care 40: 346–351. 10.2337/dc16-1295. 28031419

[aos14954-bib-0029] Broadbent DM , Wang A , Cheyne CP et al. (2021): Safety and cost‐effectiveness of individualised screening for diabetic retinopathy: the ISDR open‐label, equivalence RCT. Diabetologia 64: 56–69. 10.1007/s00125-020-05313-2. 33146763 PMC7716929

[aos14954-bib-0030] Brownlee M (2001): Biochemistry and molecular cell biology of diabetic complications. Nature 414: 813–820. 10.1038/414813a. 11742414

[aos14954-bib-0031] Brownlee M , Cerami A & Vlassara H (1988): Advanced glycosylation end products in tissue and the biochemical basis of diabetic complications. N Engl J Med 318: 1315–1321. 10.1056/nejm198805193182007. 3283558

[aos14954-bib-0032] Brownlee M , Vlassara H & Cerami A (1985): Nonenzymatic glycosylation products on collagen covalently trap low‐density lipoprotein. Diabetes 34: 938–941. 10.2337/diab.34.9.938. 4029512

[aos14954-bib-0033] Canning P , Kenny BA , Prise V et al. (2016): Lipoprotein‐associated phospholipase A2 (Lp‐PLA2) as a therapeutic target to prevent retinal vasopermeability during diabetes. Proc Natl Acad Sci USA 113: 7213–7218. 10.1073/pnas.1514213113. 27298369 PMC4932924

[aos14954-bib-0034] Celebiler Cavusoglu A , Bilgili S , Alaluf A et al. (2007): Vascular endothelial growth factor level in the serum of diabetic patients with retinopathy. Ann Ophthalmol 39: 205–208. 10.1007/s12009-007-0037-2. 18025626

[aos14954-bib-0035] Chandrasekharan UM , Siemionow M , Unsal M et al. (2007): Tumor necrosis factor α (TNF‐α) receptor‐II is required for TNF‐α–induced leukocyte‐endothelial interaction in vivo. Blood 109: 1938–1944. 10.1182/blood-2006-05-020875. 17068152 PMC1801063

[aos14954-bib-0036] Chao J , Chai K , Chen L et al. (1990): Tissue kallikrein‐binding protein is a serpin. I. Purification, characterization, and distribution in normotensive and spontaneously hypertensive rats. J Boil Chem 265: 16394–16401. 2398056

[aos14954-bib-0037] Chao J , Yin H , Yao YY , Shen B , Smith RS Jr & Chao L (2006): Novel role of kallistatin in protection against myocardial ischemia‐reperfusion injury by preventing apoptosis and inflammation. Hum Gene Ther 17: 1201–1213. 10.1089/hum.2006.17.1201. 17081080

[aos14954-bib-0038] Chaturvedi N , Fuller JH , Pokras F , Rottiers R , Papazoglou N & Aiello LP & Group, t. E. S (2001): Circulating plasma vascular endothelial growth factor and microvascular complications of Type 1 diabetes mellitus: the influence of ACE inhibition. Diabetes Med 18: 288–294. 10.1046/j.1464-5491.2001.00441.x. 11437859

[aos14954-bib-0039] Chen C , Chen X , Huang H et al. (2019): Elevated plasma and vitreous levels of leucine‐rich‐α2‐glycoprotein are associated with diabetic retinopathy progression. Acta Ophthalmol 97: 260–264. 10.1111/aos.13633. 29168314

[aos14954-bib-0040] Chen L‐C , Wang E , Tai C‐S et al. (2020): Improving the reproducibility, accuracy, and stability of an electrochemical biosensor platform for point‐of‐care use. Biosens Bioelectron 155: 112111. 10.1016/j.bios.2020.112111. 32217334

[aos14954-bib-0041] Cheung N , Mitchell P & Wong TY (2010): Diabetic retinopathy. Lancet 376: 124–136. 10.1016/s0140-6736(09)62124-3. 20580421

[aos14954-bib-0042] Choudhuri S , Dutta D , Sen A et al. (2013): Role of N‐ɛ‐ carboxy methyl lysine, advanced glycation end products and reactive oxygen species for the development of nonproliferative and proliferative retinopathy in type 2 diabetes mellitus. Molecular vision 19: 100–113. Retrieved from https://www.ncbi.nlm.nih.gov/pubmed/23378723, https://www.ncbi.nlm.nih.gov/pmc/articles/PMC3559098/. 23378723 PMC3559098

[aos14954-bib-0043] Chuang H‐S , Chen Y‐J & Cheng H‐P (2018): Enhanced diffusometric immunosensing with grafted gold nanoparticles for detection of diabetic retinopathy biomarker tumor necrosis factor‐α. Biosens Bioelectron 101: 75–83. 10.1016/j.bios.2017.10.002. 29040917

[aos14954-bib-0044] Chung JO , Park S‐Y , Cho DH , Chung DJ & Chung MY (2019): Associations between serum apolipoproteins, urinary albumin excretion rate, estimated glomerular filtration rate, and diabetic retinopathy in individuals with type 2 diabetes. Medicine 98: e15703. 10.1097/MD.0000000000015703. 31096517 PMC6531206

[aos14954-bib-0045] Claessen H , Kvitkina T , Narres M , Trautner C , Zöllner I , Bertram B & Icks A (2018): Markedly decreasing incidence of blindness in people with and without diabetes in Southern Germany. Diabetes Care 41: 478–484. 10.2337/dc17-2031. 29317450

[aos14954-bib-0046] Cook NR (2007): Use and misuse of the receiver operating characteristic curve in risk prediction. Circulation 115: 928–935. 10.1161/CIRCULATIONAHA.106.672402. 17309939

[aos14954-bib-0174] Core NDESP team . (2012). Diabetic Eye Screening Feature Based Grading Forms. Version 1.4, 1 November 2012 Software Supplier Guidance. NDESP. Available at: https://assets.publishing.service.gov.uk/government/uploads/system/uploads/attachment_data/file/402295/Feature_Based_Grading_Forms_V1_4_1Nov12_SSG.pdf. (Accessed on 30 Jun 2021).

[aos14954-bib-0047] Costagliola C , Romano V , De Tollis M et al. (2013): TNF‐alpha levels in tears: a novel biomarker to assess the degree of diabetic retinopathy. Mediators Inflamm 2013: 6. 10.1155/2013/629529. PMC3821908 24259948

[aos14954-bib-0048] Csősz É , Deák E , Kalló G , Csutak A & Tőzsér J (2017): Diabetic retinopathy: Proteomic approaches to help the differential diagnosis and to understand the underlying molecular mechanisms. J Proteomics 150: 351–358. 10.1016/j.jprot.2016.06.034. 27373871

[aos14954-bib-0049] Dan‐Brezis I , Zahavi A , Axer‐Siegel R , Nisgav Y , Dahbash M , Weinberger D , Ehrlich R & Livnat T (2020): Inflammation, angiogenesis and coagulation interplay in a variety of retinal diseases. Acta Ophthalmol 98: 10.1111/aos.14331. 31833198

[aos14954-bib-0050] Davis MD , Sheetz MJ , Aiello LP et al. (2009): Effect of ruboxistaurin on the visual acuity decline associated with long‐standing diabetic macular edema. Invest Ophthalmol Vis Sci 50: 1–4. 10.1167/iovs.08-2473. 18708615

[aos14954-bib-0051] DCCT, T. (1995): The relationship of glycemic exposure (HbA_1c_) to the risk of development and progression of retinopathy in the diabetes control and complications trial. Diabetes 44: 968–983. 10.2337/diab.44.8.968. 7622004

[aos14954-bib-0052] Derevjanik NL , Vinores SA , Xiao W‐H , Mori K , Turon T , Hudish T , Dong S & Campochiaro PA (2002): Quantitative assessment of the integrity of the blood‐retinal barrier in mice. Invest Ophthalmol Vis Sci 43: 2462–2467. 12091451

[aos14954-bib-0053] Devi KSS , Anantharamakrishnan A , Krishnan UM & Yakhmi J (2020): Chemical Sensors Based on Metal Oxides. In: Hallil H & Heidari H (eds.). Smart Sensors for Environmental and Medical Applications, Vol. 1, 1st edn. Piscataway, NJ: IEEE Press 103–127. 10.1002/9781119587422.ch6.

[aos14954-bib-0054] Devi KSS & Krishnan UM (2020): Microfluidic electrochemical immunosensor for the determination of cystatin C in human serum. Mikrochim Acta 187: 585. 10.1007/s00604-020-04503-4. 32997226

[aos14954-bib-0055] Dinesh B , Shalini Devi KS & Krishnan UM (2019): Achieving a stable high surface excess of glucose oxidase on pristine multiwalled carbon nanotubes for glucose quantification. ACS Appl Bio Mater 2: 1740–1750. 10.1021/acsabm.9b00145. 35026909

[aos14954-bib-0056] Donahue MP , Rose K , Hochstrasser D et al. (2006): Discovery of proteins related to coronary artery disease using industrial‐scale proteomics analysis of pooled plasma. Am Heart J 152: 478–485. 10.1016/j.ahj.2006.03.007. 16923417

[aos14954-bib-0057] Doublier S , Ceretto M , Lupia E , Bravo S , Bussolati B & Camussi G (2007): The proangiogenic phenotype of tumor‐derived endothelial cells is reverted by the overexpression of platelet‐activating factor acetylhydrolase. Clin Cancer Res 13: 5710–5718. 10.1158/1078-0432.Ccr-07-0412. 17908960

[aos14954-bib-0058] Drexler W & Fujimoto JG (2008): State‐of‐the‐art retinal optical coherence tomography. Prog Retin Eye Res 27: 45–88. 10.1016/j.preteyeres.2007.07.005. 18036865

[aos14954-bib-0059] Du J‐H , Li X , Li R , Xu L , Ma R‐R , Liu S‐F , Zhang Z & Sun H‐Z (2014): Elevation of serum apelin‐13 associated with proliferative diabetic retinopathy in type 2 diabetic patients. Int J Ophthalmol 7: 968–973. 10.3980/j.issn.2222-3959.2014.06.10. 25540748 PMC4270990

[aos14954-bib-0060] Duckworth W , Abraira C , Moritz T et al. (2009): Glucose control and vascular complications in veterans with type 2 diabetes. N Engl J Med 360: 129–139. 10.1056/NEJMoa0808431. 19092145

[aos14954-bib-0061] Elahy M , Baindur‐Hudson S , Cruzat VF , Newsholme P & Dass CR (2014): Mechanisms of PEDF‐mediated protection against reactive oxygen species damage in diabetic retinopathy and neuropathy. J Endocrinol 222: R129–139. 10.1530/joe-14-0065. 24928938

[aos14954-bib-0062] El‐Asrar MA , Andrawes NG , Ismail EA & Salem SM (2015): Kallistatin as a marker of microvascular complications in children and adolescents with type 1 diabetes mellitus: Relation to carotid intima media thickness. Vasc Med 20: 509–517. 10.1177/1358863x15591089. 26091968

[aos14954-bib-0063] ETDRS (1991): Grading diabetic retinopathy from stereoscopic color fundus photographs–an extension of the modified Airlie House classification. ETDRS report number 10. Early Treatment Diabetic Retinopathy Study Research Group. Ophthalmology 98(5 Suppl): 786–806. 2062513

[aos14954-bib-0065] Feige JJ , Negoescu A , Keramidas M , Souchelnitskiy S & Chambaz EM (1996): Alpha 2‐macroglobulin: a binding protein for transforming growth factor‐beta and various cytokines. Horm Res 45(3–5): 227–232. 10.1159/000184793. 8964589

[aos14954-bib-0066] Feng S , Yu H , Yu Y et al. (2018): Levels of inflammatory cytokines IL‐1β, IL‐6, IL‐8, IL‐17A, and TNF‐α in aqueous humour of patients with diabetic retinopathy. J Diabetes Res 2018: 6. 10.1155/2018/8546423. PMC5904804 29850610

[aos14954-bib-0067] Flaxman SR , Bourne RRA , Resnikoff S et al. (2017): Global causes of blindness and distance vision impairment 1990–2020: a systematic review and meta‐analysis. Lancet Global Health 5: e1221–e1234. 10.1016/S2214-109X(17)30393-5. 29032195

[aos14954-bib-0068] Foo V , Quah J , Cheung G et al. (2017): HbA1c, systolic blood pressure variability and diabetic retinopathy in Asian type 2 diabetics. J Diabetes 9: 200–207. 10.1111/1753-0407.12403. 27043025

[aos14954-bib-0069] Ford JA , Lois N , Royle P , Clar C , Shyangdan D & Waugh N (2013): Current treatments in diabetic macular oedema: systematic review and meta‐analysis. BMJ Open 3: e002269. 10.1136/bmjopen-2012-002269. PMC3612765 23457327

[aos14954-bib-0070] French K , Yerbury JJ & Wilson MRJB (2008): Protease activation of α2‐macroglobulin modulates a chaperone‐like action with broad specificity. Biochemistry 47: 1176–1185. 18171086 10.1021/bi701976f

[aos14954-bib-0071] Fujimoto M , Serada S , Suzuki K et al. (2015): Brief report: Leucine‐Rich α2‐glycoprotein as a potential biomarker for joint inflammation during anti–interleukin‐6 biologic therapy in rheumatoid arthritis. Arthrit Rheumatol 67: 2056–2060. 10.1002/art.39164 25917892

[aos14954-bib-0072] Fujiwara Y , Tagami S & Kawakami Y (1998): Circulating thrombomodulin and hematological alterations in type 2 diabetic patients with retinopathy. J Atheroscler Thromb 5: 21–28. 10077454 10.5551/jat1994.5.21

[aos14954-bib-0073] Gabbay KH (1973): The sorbitol pathway and the complications of diabetes. N Engl J Med 288: 831–836. 10.1056/nejm197304192881609. 4266466

[aos14954-bib-0074] Gao BB , Chen X , Timothy N , Aiello LP & Feener EP (2008): Characterization of the vitreous proteome in diabetes without diabetic retinopathy and diabetes with proliferative diabetic retinopathy. J Proteome Res 7: 2516–2525. 10.1021/pr800112g. 18433156

[aos14954-bib-0075] Garcia‐Ramirez M , Canals F , Hernandez C , Colome N , Ferrer C , Carrasco E , Garcia‐Arumi J & Simo R (2007): Proteomic analysis of human vitreous fluid by fluorescence‐based difference gel electrophoresis (DIGE): a new strategy for identifying potential candidates in the pathogenesis of proliferative diabetic retinopathy. Diabetologia 50: 1294–1303. 10.1007/s00125-007-0627-y. 17380318

[aos14954-bib-0076] Geraldes P & King GL (2010): Activation of protein kinase C isoforms and its impact on diabetic complications. Circ Res 106: 1319–1331. 10.1161/CIRCRESAHA.110.217117. 20431074 PMC2877591

[aos14954-bib-0077] Girolami A , Ferrari S , Cosi E , Girolami B & Lombardi AM (2018): Congenital prothrombin defects: they are not only associated with bleeding but also with thrombosis: a new classification is needed. Hematology 23: 105–110. 10.1080/10245332.2017.1359900. 28762299

[aos14954-bib-0078] Goh JKH , Cheung CY , Sim SS , Tan PC , Tan GSW & Wong TY (2016): Retinal imaging techniques for diabetic retinopathy screening. J Diabetes Sci Technol 10: 282–294. 10.1177/1932296816629491. 26830491 PMC4773981

[aos14954-bib-0079] Gold B , Merriam JE , Zernant J et al. (2006): Variation in factor B (BF) and complement component 2 (C2) genes is associated with age‐related macular degeneration. Nat Genet 38: 458–462. 10.1038/ng1750. 16518403 PMC2921703

[aos14954-bib-0080] Goldstein DE , Little RR , Lorenz RA , Malone JI , Nathan D , Peterson CM & Sacks DB (2004): Tests of glycemia in diabetes. Diabetes Care 27: 1761–1773. 10.2337/diacare.27.7.1761. 15220264

[aos14954-bib-0081] Gong Q & Su G (2017): Roles of miRNAs and long noncoding RNAs in the progression of diabetic retinopathy. Biosci Rep 37: BSR20171157. 10.1042/BSR20171157. 29074557 PMC5705777

[aos14954-bib-0082] Graham TE , Yang Q , Blüher M et al. (2006): Retinol‐binding protein 4 and insulin resistance in lean, obese, and diabetic subjects. N Engl J Med 354: 2552–2563. 10.1056/NEJMoa054862. 16775236

[aos14954-bib-0083] Gray RS , James K , Merriman J , Starkey IR , Elton RA , Clarke BF & Duncan LJ (1982): Alpha 2‐macroglobulin and proliferative retinopathy in type 1 diabetes. Horm Metab Res 14: 389–392. 10.1055/s-2007-1019026. 6182082

[aos14954-bib-0084] Green PHR , Glickman RM , Riley JW & Quinet E (1980): Human apolipoprotein A‐IV: intestinal origin and distribution in plasma. J Clin Invest 65: 911–919. 10.1172/JCI109745. 6987270 PMC434480

[aos14954-bib-0085] Grunwald JE , Ying GS , Maguire M et al. (2012): Association between retinopathy and cardiovascular disease in patients with chronic kidney disease (from the Chronic Renal Insufficiency Cohort [CRIC] Study). Am J Cardiol 110: 246–253. 10.1016/j.amjcard.2012.03.014. 22516527 PMC3383900

[aos14954-bib-0086] Guzik TJ , Mussa S , Gastaldi D , Sadowski J , Ratnatunga C , Pillai R & Channon KM (2002): Mechanisms of increased vascular superoxide production in human diabetes mellitus. Circulation 105: 1656–1662. 10.1161/01.CIR.0000012748.58444.08. 11940543

[aos14954-bib-0087] Hahr AJ & Molitch ME (2010): Diabetes, cardiovascular risk and nephropathy. Cardiol Clin 28: 467–475. 20621251 10.1016/j.ccl.2010.04.006

[aos14954-bib-0088] Hainsworth DP , Bebu I , Aiello LP et al. (2019): Risk factors for retinopathy in type 1 diabetes: The DCCT/EDIC study. Diabetes Care 42: 875–882. 10.2337/dc18-2308. 30833368 PMC6489114

[aos14954-bib-0089] Haiyashi Y , Makino H & Ota Z (1992): Serum and urinary concentrations of type IV collagen and laminin as a marker of microangiopathy in diabetes. Diabet Med 9: 366–370. 10.1111/j.1464-5491.1992.tb01798.x. 1600709

[aos14954-bib-0090] Hakansson K , Huh C , Grubb A , Karlsson S & Abrahamson M (1996): Mouse and rat cystatin C: Escherichia coli production, characterization and tissue distribution. Comp Biochem Physiol B Biochem Mol Biol 114: 303–311. 8761177 10.1016/0305-0491(96)00025-9

[aos14954-bib-0091] Hase K , Kanda A , Hirose I , Noda K & Ishida S (2017): Systemic factors related to soluble (pro)renin receptor in plasma of patients with proliferative diabetic retinopathy. PLoS One 12: e0189696. 10.1371/journal.pone.0189696. 29240802 PMC5730163

[aos14954-bib-0092] Hatcher HC , Ma JX , Chao J , Chao L & Ottlecz A (1997): Kallikrein‐binding protein levels are reduced in the retinas of streptozotocin‐induced diabetic rats. Invest Ophthalmol Vis Sci 38: 658–664. 9071220

[aos14954-bib-0093] He R , Shen J , Zhao J , Zeng H , Li L , Zhao J , Liu F & Jia W (2013): High cystatin C levels predict severe retinopathy in type 2 diabetes patients. Eur J Epidemiol 28: 775–778. 10.1007/s10654-013-9839-2. 23918210

[aos14954-bib-0094] Heiser M , Hutter‐Paier B , Jerkovic L , Pfragner R , Windisch M , Becker‐Andre M & Dieplinger H (2002): Vitamin E binding protein A famin Protects Neuronal Cells in vitro. In: Jellinger KA & Schmidt R (eds.). Ageing and Dementia Current and Future Concepts. Journal of Neural Transmission. Supplementa, Vol. 62. Vienna: Springer, pp. 337–345. 10.1007/978-3-7091-6139-5_32. 12456077

[aos14954-bib-0095] Hermann JM , Hammes H‐P , Rami‐Merhar B , Rosenbauer J , Schütt M , Siegel E , Holl RW & on behalf of the, D. P. V. I. t. G. B. C. N. D. M (2014): HbA1c variability as an independent risk factor for diabetic retinopathy in type 1 diabetes: A German/Austrian multicenter analysis on 35,891 patients. PLoS One 9: e91137. 10.1371/journal.pone.0091137. 24609115 PMC3946653

[aos14954-bib-0096] Hertle E , Arts IC , van der Kallen CJ , Feskens EJ , Schalkwijk CG , Stehouwer CD & van Greevenbroek MM (2016): The alternative complement pathway is longitudinally associated with adverse cardiovascular outcomes. The CODAM study. Thromb Haemost 115: 446–457. 10.1160/th15-05-0439. 26446431

[aos14954-bib-0097] Hirata K & Kubo K (2004): Relationship between blood levels of *N*‐carboxymethyl‐lysine and pentosidine and the severity of microangiopathy in type 2 diabetes. Endocr J 51: 537–544. 10.1507/endocrj.51.537. 15644571

[aos14954-bib-0098] Hirsch IB & Brownlee M (2010): Beyond hemoglobin A1c—need for additional markers of risk for diabetic microvascular complications. JAMA 303: 2291–2292. 10.1001/jama.2010.785. 20530784

[aos14954-bib-0099] Honda H , Fujimoto M , Miyamoto S et al. (2016): Sputum leucine‐rich alpha‐2 glycoprotein as a marker of airway inflammation in asthma. PLoS One 11: e0162672. 27611322 10.1371/journal.pone.0162672 PMC5017625

[aos14954-bib-0100] Hoo ZH , Candlish J & Teare D (2017): What is an ROC curve? Emerg Med J 34: 357–359. 10.1136/emermed-2017-206735. 28302644

[aos14954-bib-0101] Hu A , Luo Y , Li T , Guo X , Ding X , Zhu X , Wang X & Tang S (2012): Low serum apolipoprotein A1/B ratio is associated with proliferative diabetic retinopathy in type 2 diabetes. Graefes Arch Clin Exp Ophthalmol 250: 957–962. 10.1007/s00417-011-1855-x. 22327732

[aos14954-bib-0102] Huang C , Fisher KP , Hammer SS , Navitskaya S , Blanchard GJ & Busik JV (2018): Plasma exosomes contribute to microvascular damage in diabetic retinopathy by activating the classical complement pathway. Diabetes 67: 1639–1649. 10.2337/db17-1587. 29866771 PMC6054433

[aos14954-bib-0103] Idiris A , Ohtsubo K‐I , Yoza K‐I , Osada T , Nakamichi N , Matsumura T & Ikai AJJ o. p. c. (2003): Molecular cloning and structural characterization of the hagfish proteinase inhibitor of the alpha‐2‐macroglobulin family. J Protein Chem 22: 89–98. 12739901 10.1023/a:1023076029496

[aos14954-bib-0064] International Diabetes Federation . (2019): IDF Diabetes Atlas. 9th edn. Brussels: IDF. Available at: https://www.diabetesatlas.org. (Accessed on 30 Jun 2021).

[aos14954-bib-0104] Ioannidis JPA & Bossuyt PMM (2017): Waste, leaks, and failures in the biomarker pipeline. Clin Chem 63: 963–972. 10.1373/clinchem.2016.254649. 28270433

[aos14954-bib-0105] Jacqueminet S , Ben Abdesselam O , Chapman MJ , Nicolay N , Foglietti MJ , Grimaldi A & Beaudeux JL (2006): Elevated circulating levels of matrix metalloproteinase‐9 in type 1 diabetic patients with and without retinopathy. Clin Chim Acta 367: 103–107. 10.1016/j.cca.2005.11.029. 16426593

[aos14954-bib-0106] Jain A , Saxena S , Khanna VK , Shukla RK & Meyer CH (2013): Status of serum VEGF and ICAM‐1 and its association with external limiting membrane and inner segment‐outer segment junction disruption in type 2 diabetes mellitus. Mol Vis 19: 1760–1768. Retrieved from https://www.ncbi.nlm.nih.gov/pubmed/23922493 https://www.ncbi.nlm.nih.gov/pmc/articles/PMC3733909/. 23922493 PMC3733909

[aos14954-bib-0107] James K , Merriman J , Gray RS , Duncan LJ & Herd R (1980): Serum alpha 2‐macroglobulin levels in diabetes. J Clin Pathol 33: 163–166. Retrieved from http://www.ncbi.nlm.nih.gov/pubmed/6154066. 6154066 10.1136/jcp.33.2.163 PMC1146013

[aos14954-bib-0108] Jampol LM , Glassman AR & Sun J (2020): Evaluation and care of patients with diabetic retinopathy. N Engl J Med 382: 1629–1637. 10.1056/NEJMra1909637. 32320570

[aos14954-bib-0109] Jenkins AJ , Joglekar MV , Hardikar AA , Keech AC , O'Neal DN & Januszewski AS (2015): Biomarkers in diabetic retinopathy. Rev Diabetic Stud RDS 12(1–2): 159–195. 10.1900/RDS.2015.12.159. 26676667 PMC5397989

[aos14954-bib-0110] Jenkins AJ , McBride JD , Januszewski AS et al. (2010): Increased serum kallistatin levels in type 1 diabetes patients with vascular complications. J Angiogenes Res 2: 19. 10.1186/2040-2384-2-19. 20860825 PMC2954956

[aos14954-bib-0111] Jenkins A , Zhang S , Rowley K et al. (2007): Increased serum pigment epithelium‐derived factor is associated with microvascular complications, vascular stiffness and inflammation in Type 1 diabetes 1. Diabet Med 24: 1345–1351. 17971181 10.1111/j.1464-5491.2007.02281.x

[aos14954-bib-0112] Jeon YK , Kim MR , Huh JE et al. (2011): Cystatin C as an early biomarker of nephropathy in patients with type 2 diabetes. J Korean Med Sci 26: 258–263. 10.3346/jkms.2011.26.2.258. 21286018 PMC3031011

[aos14954-bib-0113] Jerkovic L , Voegele AF , Chwatal S et al. (2005): Afamin is a novel human vitamin E‐binding glycoprotein characterization and in vitro expression. J Proteome Res 4: 889–899. 15952736 10.1021/pr0500105

[aos14954-bib-0114] Jin J , Min H , Kim SJ , Oh S , Kim K , Yu HG , Park T & Kim Y (2016): Development of diagnostic biomarkers for detecting diabetic retinopathy at early stages using quantitative proteomics. J Diabetes Res 2016: 22. 10.1155/2016/6571976. PMC4657408 26665153

[aos14954-bib-0115] Joussen AM , Poulaki V , Qin W et al. (2002): Retinal vascular endothelial growth factor induces intercellular adhesion molecule‐1 and endothelial nitric oxide synthase expression and initiates early diabetic retinal leukocyte adhesion in vivo. Am J Pathol 160: 501–509. 10.1016/S0002-9440(10)64869-9. 11839570 PMC1850650

[aos14954-bib-0116] Karakaya M & Hacisoftaoglu RE (2020): Comparison of smartphone‐based retinal imaging systems for diabetic retinopathy detection using deep learning. BMC Bioinf 21(Suppl 4): 259. 10.1186/s12859-020-03587-2. PMC7336606 32631221

[aos14954-bib-0117] Katan M , Moon YP , Paik MC , Wolfert RL , Sacco RL & Elkind MS (2014): Lipoprotein‐associated phospholipase A2 is associated with atherosclerotic stroke risk: the Northern Manhattan Study. PLoS One 9: e83393. 10.1371/journal.pone.0083393. 24416164 PMC3886969

[aos14954-bib-0118] Kaviarasan K , Jithu M , Arif Mulla M , Sharma T , Sivasankar S , Das UN & Angayarkanni N (2015): Low blood and vitreal BDNF, LXA4 and altered Th1/Th2 cytokine balance are potential risk factors for diabetic retinopathy. Metabolism 64: 958–966. 10.1016/j.metabol.2015.04.005. 26004392

[aos14954-bib-0119] Kerkeni M , Saïdi A , Bouzidi H , Yahya SB & Hammami M (2012): Elevated serum levels of AGEs, sRAGE, and pentosidine in Tunisian patients with severity of diabetic retinopathy. Microvasc Res 84: 378–383. 10.1016/j.mvr.2012.07.006. 22835520

[aos14954-bib-0120] Kilpatrick ES , Rigby AS & Atkin SL (2008): A1C variability and the risk of microvascular complications in type 1 diabetes. Diabetes Care 31: 2198–2202. 10.2337/dc08-0864. 18650371 PMC2571045

[aos14954-bib-0121] Kim HJ , Byun DW , Suh K , Yoo MH & Park HK (2018): Association between serum cystatin C and vascular complications in type 2 diabetes mellitus without nephropathy. Diabetes Metab J 42: 513–518. 10.4093/dmj.2018.0006. 30398035 PMC6300438

[aos14954-bib-0122] Kim HU , Park SP & Kim Y‐K (2021): Long‐term HbA1c variability and the development and progression of diabetic retinopathy in subjects with type 2 diabetes. Sci Rep 11: 4731. 10.1038/s41598-021-84150-8. 33637847 PMC7910456

[aos14954-bib-0123] Kim K , Kim SJ , Han D , Jin J , Yu J , Park KS , Yu HG & Kim Y (2013): Verification of multimarkers for detection of early stage diabetic retinopathy using multiple reaction monitoring. J Proteome Res 12: 1078–1089. 10.1021/pr3012073. 23368427

[aos14954-bib-0124] Kim T , Kim SJ , Kim K , Kang UB , Lee C , Park KS , Yu HG & Kim Y (2007): Profiling of vitreous proteomes from proliferative diabetic retinopathy and nondiabetic patients. Proteomics 7: 4203–4215. 10.1002/pmic.200700745. 17955474

[aos14954-bib-0125] Klein BEK , Knudtson MD , Tsai MY & Klein R (2009): The relation of markers of inflammation and endothelial dysfunction to the prevalence and progression of diabetic retinopathy: wisconsin epidemiologic study of diabetic retinopathyinflammation markers and diabetic retinopathy. JAMA Ophthalmol 127: 1175–1182. 10.1001/archophthalmol.2009.172. PMC2746055 19752427

[aos14954-bib-0126] Klein RL , McHenry MB , Lok KH et al. (2005): Apolipoprotein C‐III protein concentrations and gene polymorphisms in Type 1 diabetes: associations with microvascular disease complications in the DCCT/EDIC cohort. J Diabetes Complicat 19: 18–25. 10.1016/j.jdiacomp.2004.04.005. 15642486

[aos14954-bib-0127] Kobe B & Kajava AV (2001): The leucine‐rich repeat as a protein recognition motif. Curr Opin Struct Biol 11: 725–732. 11751054 10.1016/s0959-440x(01)00266-4

[aos14954-bib-0128] Kollerits B , Lamina C , Huth C et al. (2017): Plasma concentrations of afamin are associated with prevalent and incident type 2 diabetes: a pooled analysis in more than 20,000 individuals. Diabetes Care 40: 1386–1393. 10.2337/dc17-0201. 28877915

[aos14954-bib-0129] Kotajima N , Kanda T , Yuuki N , Kimura T , Kishi S , Fukumura Y , Tamura J & Kobayashi I (2001): Type IV collagen serum and vitreous fluid levels in patients with diabetic retinopathy. J Int Med Res 29: 292–296. 10.1177/147323000102900405. 11675902

[aos14954-bib-0130] Kou X , Tong L , Shen Y et al. (2020): Smartphone‐assisted robust enzymes@MOFs‐based paper biosensor for point‐of‐care detection. Biosens Bioelectron 156: 112095. 10.1016/j.bios.2020.112095. 32174563

[aos14954-bib-0131] Kowluru RA & Mishra M (2017): Chapter Three ‐ Regulation of Matrix Metalloproteinase in the Pathogenesis of Diabetic Retinopathy. In: Khalil RA (ed.). Progress in Molecular Biology and Translational Science, Vol. 148., Academic Press, 67–85. 28662829 10.1016/bs.pmbts.2017.02.004

[aos14954-bib-0132] Koya D & King GL (1998): Protein kinase C activation and the development of diabetic complications. Diabetes 47: 859–866. 10.2337/diabetes.47.6.859. 9604860

[aos14954-bib-0133] Kratzer I , Bernhart E , Wintersperger A et al. (2009): Afamin is synthesized by cerebrovascular endothelial cells and mediates α‐tocopherol transport across an in vitro model of the blood–brain barrier. J Neurochem 108: 707–718. 19046407 10.1111/j.1471-4159.2008.05796.x PMC3064965

[aos14954-bib-0134] Kronenberg F , Kollerits B , Kiechl S et al. (2014): Plasma concentrations of afamin are associated with the prevalence and development of metabolic syndrome. Circ Cardiovasc Genet 7: 822–829. 10.1161/circgenetics.113.000654. 25176938

[aos14954-bib-0135] Kwon J‐W , Choi JA & Jee D (2016): Matrix metalloproteinase‐1 and matrix metalloproteinase‐9 in the aqueous humor of diabetic macular edema patients. PLoS One 11: e0159720. 10.1371/journal.pone.0159720. 27467659 PMC4965102

[aos14954-bib-0136] LaMarre J , Wollenberg GK , Gonias SL & Hayes MA (1991): Cytokine binding and clearance properties of proteinase‐activated alpha 2‐macroglobulins. Lab Invest 65: 3–14. 1712874

[aos14954-bib-0137] Leasher JL , Bourne RRA , Flaxman SR et al. (2016): Global estimates on the number of people blind or visually impaired by diabetic retinopathy: a meta‐analysis from 1990 to 2010. Diabetes Care 39: 1643–1649. 10.2337/dc15-2171. 27555623

[aos14954-bib-0138] Lee IK , Park KY , Oh HK , Park RW & Jo JS (1994): Plasma type IV collagen and fibronectin concentrations in diabetic patients with microangiopathy. J Korean Med Sci 9: 341–346. 10.3346/jkms.1994.9.4.341. 7848583 PMC3054096

[aos14954-bib-0139] Lee WJ , Sobrin L , Lee MJ , Kang MH , Seong M & Cho H (2014): The relationship between diabetic retinopathy and diabetic nephropathy in a population‐based study in Korea (KNHANES V‐2, 3). Invest Ophthalmol Vis Sci 55: 6547–6553. 10.1167/iovs.14-15001. 25205863

[aos14954-bib-0140] Li J‐Y , Chen X‐X , Lu X‐H , Zhang C‐B , Shi Q‐P & Feng L (2018): Elevated RBP4 plasma levels were associated with diabetic retinopathy in type 2 diabetes. Biosci Rep 38: BSR20181100. 10.1042/BSR20181100. 30135138 PMC6131341

[aos14954-bib-0141] Li P , Bledsoe G , Yang ZR , Fan H , Chao L & Chao J (2014): Human kallistatin administration reduces organ injury and improves survival in a mouse model of polymicrobial sepsis. Immunology 142: 216–226. 10.1111/imm.12242. 24467264 PMC4008229

[aos14954-bib-0142] Li S , Fu X‐A , Zhou X‐F , Chen Y‐Y & Chen W‐Q (2012): Angiogenesis‐related cytokines in serum of proliferative diabetic retinopathy patients before and after vitrectomy. Int J Ophthalmol 5: 726. 23275908 10.3980/j.issn.2222-3959.2012.06.14 PMC3530816

[aos14954-bib-0143] Li X , Miyajima M , Jiang C & Arai H (2007): Expression of TGF‐βs and TGF‐β type II receptor in cerebrospinal fluid of patients with idiopathic normal pressure hydrocephalus. Neurosci Lett 413: 141–144. 17194537 10.1016/j.neulet.2006.11.039

[aos14954-bib-0144] Li Z‐Z , Lu X‐Z , Liu J‐B & Chen L (2010): Serum retinol‐binding protein 4 levels in patients with diabetic retinopathy. J Int Med Res 38: 95–99. 10.1177/147323001003800111. 20233518

[aos14954-bib-0145] Lichenstein HS , Lyons DE , Wurfel MM et al. (1994): Afamin is a new member of the albumin, alpha‐fetoprotein, and vitamin D‐binding protein gene family. J Biol Chem 269: 18149–18154. 7517938

[aos14954-bib-0146] Liew G , Michaelides M & Bunce C (2014): A comparison of the causes of blindness certifications in England and Wales in working age adults (16–64 years), 1999–2000 with 2009–2010. BMJ Open 4: e004015. 10.1136/bmjopen-2013-004015. PMC3927710 24525390

[aos14954-bib-0147] Liew G , Wong VW & Ho IV (2017): Mini review: changes in the incidence of and progression to proliferative and sight‐threatening diabetic retinopathy over the last 30 years. Ophthalmic Epidemiol 24: 73–80. 10.1080/09286586.2016.1259638. 28102748

[aos14954-bib-0148] Lind M , Pivodic A , Svensson A‐M , Ólafsdóttir AF , Wedel H & Ludvigsson J (2019): HbA_1c_ level as a risk factor for retinopathy and nephropathy in children and adults with type 1 diabetes: Swedish population based cohort study. BMJ 366: l4894. 10.1136/bmj.l4894. 31462492 PMC6712507

[aos14954-bib-0149] Liu J & Feener EP (2013): Plasma kallikrein‐kinin system and diabetic retinopathy. Biol Chem 394: 319–328. 10.1515/hsz-2012-0316. 23362193 PMC4844060

[aos14954-bib-0150] Liu X , Zhang B , McBride JD , Zhou K , Lee K , Zhou Y , Liu Z & Ma JX (2013): Antiangiogenic and antineuroinflammatory effects of kallistatin through interactions with the canonical Wnt pathway. Diabetes 62: 4228–4238. 10.2337/db12-1710. 23884893 PMC3837048

[aos14954-bib-0151] Liu X , Zhao P , Tang S et al. (2010): Association study of complement factor H, C2, CFB, and C3 and age‐related macular degeneration in a Han Chinese population. Retina 30: 1177–1184. 10.1097/IAE.0b013e3181cea676. 20523265

[aos14954-bib-0152] Lorenzi M (2007): The polyol pathway as a mechanism for diabetic retinopathy: attractive, elusive, and resilient. Exp Diabetes Res 2007: 1–10. 10.1155/2007/61038. PMC1950230 18224243

[aos14954-bib-0153] Lu CH , Lin ST , Chou HC , Lee YR & Chan HL (2013): Proteomic analysis of retinopathy‐related plasma biomarkers in diabetic patients. Arch Biochem Biophys 529: 146–156. 10.1016/j.abb.2012.11.004. 23220024

[aos14954-bib-0154] Lynch JJ , Ferro TJ , Blumenstock FA , Brockenauer AM & Malik AB (1990): Increased endothelial albumin permeability mediated by protein kinase C activation. J Clin Invest 85: 1991–1998. 10.1172/JCI114663. 2347922 PMC296668

[aos14954-bib-0155] Lyons TJ & Basu A (2012): Biomarkers in diabetes: hemoglobin A1c, vascular and tissue markers. Transl Res 159: 303–312. 10.1016/j.trsl.2012.01.009. 22424433 PMC3339236

[aos14954-bib-0156] Ma JX , King LP , Yang Z , Crouch RK , Chao L & Chao J (1996): Kallistatin in human ocular tissues: reduced levels in vitreous fluids from patients with diabetic retinopathy. Curr Eye Res 15: 1117–1123. 8950506 10.3109/02713689608995143

[aos14954-bib-0157] Mansour SE , Browning DJ , Wong K , Flynn HW Jr & Bhavsar AR (2020): The evolving treatment of diabetic retinopathy. Clin Ophthalmol (Auckland N.Z.), 14: 653–678. 10.2147/OPTH.S236637. PMC7061411 32184554

[aos14954-bib-0158] Maragoudakis ME , Tsopanoglou NE & Andriopoulou P (2002): Mechanism of thrombin‐induced angiogenesis. Biochem Soc Trans 30: 173–177. 12023846 10.1042/

[aos14954-bib-0159] Martinez B & Peplow PV (2019): MicroRNAs as biomarkers of diabetic retinopathy and disease progression. Neural Regen Res 14: 1858–1869. 10.4103/1673-5374.259602. 31290435 PMC6676865

[aos14954-bib-0160] Maxwell PR , Timms PM , Chandran S & Gordon D (2001): Peripheral blood level alterations of TIMP‐1, MMP‐2 and MMP‐9 in patients with Type 1 diabetes. Diabet Med 18: 777–780. 10.1046/j.1464-5491.2001.00542.x. 11678966

[aos14954-bib-0161] McAuley AK , Sanfilippo PG , Hewitt AW , Liang H , Lamoureux E , Wang JJ & Connell PP (2014): Vitreous biomarkers in diabetic retinopathy: a systematic review and meta‐analysis. J Diabetes Complications 28: 419–425. 24630762 10.1016/j.jdiacomp.2013.09.010

[aos14954-bib-0162] McBride JD , Jenkins AJ , Liu X et al. (2014): Elevated circulation levels of an antiangiogenic SERPIN in patients with diabetic microvascular complications impair wound healing through suppression of wnt signaling. J Invest Dermatol 134: 1725–1734. 10.1038/jid.2014.40. 24463424 PMC4065799

[aos14954-bib-0163] McLaughlin T , Ackerman SE , Shen L & Engleman E (2017): Role of innate and adaptive immunity in obesity‐associated metabolic disease. J Clin Invest 127: 5–13. 10.1172/jci88876. 28045397 PMC5199693

[aos14954-bib-0164] Mendivil CO , Zheng C , Furtado J , Lel J & Sacks FM (2010): Metabolism of very‐low‐density lipoprotein and low‐density lipoprotein containing apolipoprotein C‐III and not other small apolipoproteins. Arterioscler Thromb Vasc Biol 30: 239–245. 10.1161/atvbaha.109.197830. 19910636 PMC2818784

[aos14954-bib-0165] Miyake T , Gahara Y , Nakayama M , Yamada H , Uwabe K & Kitamura T (1996): Up‐regulation of cystatin C by microglia in the rat facial nucleus following axotomy. Brain Res Mol Brain Res 37(1–2): 273–282. 8738161 10.1016/0169-328x(95)00337-r

[aos14954-bib-0166] Moosaie F , Davatgari RM , Firouzabadi FD et al. (2020): Lipoprotein(a) and apolipoproteins as predictors for diabetic retinopathy and its severity in adults with type 2 diabetes: a case‐cohort study. Can J Diabetes 44: 414–421. 10.1016/j.jcjd.2020.01.007. 32205075

[aos14954-bib-0167] Moreno‐Navarrete JM , Martinez‐Barricarte R , Catalan V et al. (2010): Complement factor H is expressed in adipose tissue in association with insulin resistance. Diabetes 59: 200–209. 10.2337/db09-0700. 19833879 PMC2797922

[aos14954-bib-0168] Mullins ES , Kombrinck KW , Talmage KE et al. (2009): Genetic elimination of prothrombin in adult mice is not compatible with survival and results in spontaneous hemorrhagic events in both heart and brain. Blood 113: 696–704. 10.1182/blood-2008-07-169003. 18927430 PMC2628376

[aos14954-bib-0169] Mussap M & Plebani M (2004): Biochemistry and clinical role of human cystatin C. Crit Rev Clin Lab Sci 41(5–6): 467–550. 10.1080/10408360490504934. 15603510

[aos14954-bib-0170] Natarajan S , Jain A , Krishnan R , Rogye A & Sivaprasad S (2019): Diagnostic accuracy of community‐based diabetic retinopathy screening with an offline artificial intelligence system on a smartphone. JAMA Ophthalmol 137: 1182–1188. 10.1001/jamaophthalmol.2019.2923. 31393538 PMC6692680

[aos14954-bib-0171] Nath M , Halder N & Velpandian T (2017): Circulating biomarkers in glaucoma, age‐related macular degeneration, and diabetic retinopathy. Indian J Ophthalmol 65: 191–197. 10.4103/ijo.IJO_866_16. 28440247 PMC5426123

[aos14954-bib-0172] Navarro S , Debili N , Bernaudin JF , Vainchenker W & Doly J (1989): Regulation of the expression of IL‐6 in human monocytes. J Immunol 142: 4339–4345. Retrieved from https://www.jimmunol.org/content/jimmunol/142/12/4339.full.pdf. 2786029

[aos14954-bib-0173] Nderitu P , do Rio JMN & Rasheed R et al. (2021): Deep learning for gradability classification of handheld, non‐mydriatic retinal images. Sci Rep 11: 9469. 10.1038/s41598-021-89027-4. 33947946 PMC8096843

[aos14954-bib-0175] Nicoloff G , Baydanoff S , Stanimirova N , Petrova C & Christova P (2001): Detection of serum collagen type IV in children with type 1 (insulin‐dependent) diabetes mellitus – a longitudinal study. Pediatric Diabetes 2: 184–190. 10.1034/j.1399-5448.2001.20408.x. 15016185

[aos14954-bib-0176] Nordwall M , Abrahamsson M , Dhir M , Fredrikson M , Ludvigsson J & Arnqvist HJ (2015): Impact of HbA_1c_, followed from onset of type 1 diabetes, on the development of severe retinopathy and nephropathy: the VISS study (Vascular Diabetic Complications in Southeast Sweden). Diabetes Care 38: 308–315. 10.2337/dc14-1203. 25510400

[aos14954-bib-0177] O’Donnell LC , Druhan LJ & Avalos BR (2002): Molecular characterization and expression analysis of leucine‐rich α2‐glycoprotein, a novel marker of granulocytic differentiation. J Leukocyte Biol 72: 478–485. 12223515

[aos14954-bib-0178] Oei HH , van der Meer IM , Hofman A , Koudstaal PJ , Stijnen T , Breteler MM & Witteman JC (2005): Lipoprotein‐associated phospholipase A2 activity is associated with risk of coronary heart disease and ischemic stroke: the Rotterdam Study. Circulation 111: 570–575. 10.1161/01.Cir.0000154553.12214.Cd. 15699277

[aos14954-bib-0179] Olsen T & Blomhoff R (2020): Retinol, retinoic acid, and retinol‐binding protein 4 are differentially associated with cardiovascular disease, type 2 diabetes, and obesity: an overview of human studies. Adv Nutr 11: 644–666. 10.1093/advances/nmz131. 31868199 PMC7231588

[aos14954-bib-0180] Onat A , Hergenc G , Ayhan E , Ugur M , Kaya H , Tuncer M & Can G (2009): Serum apolipoprotein C‐III in high‐density lipoprotein: a key diabetogenic risk factor in Turks. Diabet Med 26: 981–988. 10.1111/j.1464-5491.2009.02814.x. 19900229

[aos14954-bib-0181] Ozturk BT , Bozkurt B , Kerimoglu H , Okka M , Kamis U & Gunduz K (2009): Effect of serum cytokines and VEGF levels on diabetic retinopathy and macular thickness. Molecular vision 15: 1906–1914. Retrieved from https://www.ncbi.nlm.nih.gov/pubmed/19784389 https://www.ncbi.nlm.nih.gov/pmc/articles/PMC2751798/. 19784389 PMC2751798

[aos14954-bib-0182] Palm DE , Knuckey NW , Primiano MJ , Spangenberger AG & Johanson CE (1995): Cystatin C, a protease inhibitor, in degenerating rat hippocampal neurons following transient forebrain ischemia. Brain Res 691(1–2): 1–8. 8590041 10.1016/0006-8993(95)00520-z

[aos14954-bib-0183] Paraoan L , Hiscott P , Gosden C & Grierson I (2010): Cystatin C in macular and neuronal degenerations: implications for mechanism(s) of age‐related macular degeneration. Vis Res 50: 737–742. 10.1016/j.visres.2009.10.022. 19917302

[aos14954-bib-0184] Peeters SA , Engelen L , Buijs J , Chaturvedi N , Fuller JH , Schalkwijk CG , Stehouwer CD & Group, E. P. C. S (2015): Plasma levels of matrix metalloproteinase‐2, ‐3, ‐10, and tissue inhibitor of metalloproteinase‐1 are associated with vascular complications in patients with type 1 diabetes: the EURODIAB Prospective Complications Study. Cardiovasc Diabetol 14: 31. 10.1186/s12933-015-0195-2. 25848912 PMC4355971

[aos14954-bib-0185] Pirzada M & Altintas Z (2019): Nanomaterials for healthcare biosensing applications. Sensors 19: 5311. 10.3390/s19235311. 31810313 PMC6928990

[aos14954-bib-0186] Pusparajah P , Lee L‐H & Abdul Kadir K (2016): Molecular markers of diabetic retinopathy: potential screening tool of the future? Front Physiol 7: 200. 10.3389/fphys.2016.00200. 27313539 PMC4887489

[aos14954-bib-0187] Raffort J , Hinault C , Dumortier O & Van Obberghen E (2015): Circulating microRNAs and diabetes: potential applications in medical practice. Diabetologia 58: 1978–1992. 10.1007/s00125-015-3680-y. 26155747

[aos14954-bib-0188] Rahbar S (2005): The discovery of glycated hemoglobin: a major event in the study of nonenzymatic chemistry in biological systems. Ann N Y Acad Sci 1043: 9–19. 10.1196/annals.1333.002. 16037217

[aos14954-bib-0189] Rajalakshmi R , Shanthi Rani CS , Venkatesan U et al. (2020): Correlation between markers of renal function and sight‐threatening diabetic retinopathy in type 2 diabetes: a longitudinal study in an Indian clinic population. BMJ Open Diabetes Res Care 8: e001325. 10.1136/bmjdrc-2020-001325. PMC7265015 32475840

[aos14954-bib-0190] Rao PV , Reddy AP , Lu X , Dasari S , Krishnaprasad A , Biggs E , Roberts CT Jr , Nagalla SRJJ o. p. r. (2009): Proteomic identification of salivary biomarkers of type‐2 diabetes. J Proteome Res 8: 239–245. 19118452 10.1021/pr8003776

[aos14954-bib-0191] Rathnakumar K , Savant S , Giri H et al. (2016): Angiopoietin‐2 mediates thrombin‐induced monocyte adhesion and endothelial permeability. J Thromb Haemost 14: 1655–1667. 10.1111/jth.13376. 27241812

[aos14954-bib-0192] Ricklin D , Hajishengallis G , Yang K & Lambris JD (2010): Complement: a key system for immune surveillance and homeostasis. Nat Immunol 11: 785–797. 10.1038/ni.1923. 20720586 PMC2924908

[aos14954-bib-0193] Rifai N , Gillette MA & Carr SA (2006): Protein biomarker discovery and validation: the long and uncertain path to clinical utility. Nat Biotechnol 24: 971–983. 10.1038/nbt1235. 16900146

[aos14954-bib-0194] Romano M , Sironi M , Toniatti C et al. (1997): Role of IL‐6 and its soluble receptor in induction of chemokines and leukocyte recruitment. Immunity 6: 315–325. 10.1016/S1074-7613(00)80334-9. 9075932

[aos14954-bib-0195] Romero P , Salvat M , Fernández J , Baget M & Martinez I (2007): Renal and retinal microangiopathy after 15 years of follow‐up study in a sample of Type 1 diabetes mellitus patients. J Diabetes Complications 21: 93–100. 10.1016/j.jdiacomp.2006.04.001. 17331857

[aos14954-bib-0196] Roy MS , Janal MN , Crosby J & Donnelly R (2013): Inflammatory biomarkers and progression of diabetic retinopathy in African Americans with type 1 diabetes. Invest Ophthalmol Vis Sci 54: 5471–5480. 10.1167/iovs.13-12212. 23847308 PMC3743457

[aos14954-bib-0197] Rusling JF , Kumar CV , Gutkind JS & Patel V (2010): Measurement of biomarker proteins for point‐of‐care early detection and monitoring of cancer. Analyst 135: 2496–2511. 10.1039/c0an00204f. 20614087 PMC2997816

[aos14954-bib-0198] Safi H , Safi S , Hafezi‐Moghadam A & Ahmadieh H (2018): Early detection of diabetic retinopathy. Surv Ophthalmol 63: 601–608. 10.1016/j.survophthal.2018.04.003. 29679616

[aos14954-bib-0199] Safi SZ , Qvist R , Kumar S , Batumalaie K & Ismail ISB (2014): Molecular mechanisms of diabetic retinopathy, general preventive strategies, and novel therapeutic targets. BioMed Res Int 2014: 801269. 10.1155/2014/801269. 25105142 PMC4106080

[aos14954-bib-0200] Saito K , Tanaka T , Kanda H , Ebisuno Y , Izawa D , Kawamoto S , Okubo K & Miyasaka M (2002): Gene expression profiling of mucosal addressin cell adhesion molecule‐1+ high endothelial venule cells (HEV) and identification of a leucine‐rich HEV glycoprotein as a HEV marker. J Immunol 168: 1050–1059. 11801638 10.4049/jimmunol.168.3.1050

[aos14954-bib-0201] Salman AG , Mansour DEAA , Swelem AHA , Al‐Zawahary WMAR & Radwan AA (2009): Pentosidine – A new biochemical marker in diabetic retinopathy. Ophthalmic Res 42: 96–98. 10.1159/000225661. 19546600

[aos14954-bib-0202] Saltiel AR & Olefsky JM (2017): Inflammatory mechanisms linking obesity and metabolic disease. J Clin Invest 127: 1–4. 10.1172/jci92035. 28045402 PMC5199709

[aos14954-bib-0203] Sasongko MB , Wong TY , Nguyen TT , Kawasaki R , Jenkins A , Shaw J & Wang JJ (2011): Serum apolipoprotein AI and B are stronger biomarkers of diabetic retinopathy than traditional lipids. Diabetes Care 34: 474–479. 10.2337/dc10-0793. 21270203 PMC3024371

[aos14954-bib-0204] Schram MT , Chaturvedi N , Schalkwijk CG , Fuller JH , Stehouwer CDA & Group, E. P. C. S (2005): Markers of inflammation are cross‐sectionally associated with microvascular complications and cardiovascular disease in type 1 diabetes—the EURODIAB Prospective Complications Study. Diabetologia 48: 370–378. 10.1007/s00125-004-1628-8. 15692810

[aos14954-bib-0205] Schram MT , Chaturvedi N , Schalkwijk C , Giorgino F , Ebeling P , Fuller JH & Stehouwer CD (2003): Vascular risk factors and markers of endothelial function as determinants of inflammatory markers in type 1 diabetes. The EURODIAB Prospective Complications Study. Diabetes Care 26: 2165–2173. 10.2337/diacare.26.7.2165. 12832330

[aos14954-bib-0206] Sethi RS (1994): Transducer aspects of biosensors. Biosens Bioelectron 9: 243–264.

[aos14954-bib-0207] Shahulhameed S , Vishwakarma S , Chhablani J , Tyagi M , Pappuru RR , Jakati S , Chakrabarti S & Kaur I (2020): A Systematic investigation on complement pathway activation in diabetic retinopathy. Front Immunol 11: 154. 10.3389/fimmu.2020.00154. 32117292 PMC7026189

[aos14954-bib-0208] Shalini Devi KS , Sasya M & Krishnan UM (2020): Emerging vistas on electrochemical detection of diabetic retinopathy biomarkers. TrAC Trends Anal Chem 125: 115838. 10.1016/j.trac.2020.115838.

[aos14954-bib-0209] Shen B , Gao L , Hsu YT , Bledsoe G , Hagiwara M , Chao L & Chao J (2010): Kallistatin attenuates endothelial apoptosis through inhibition of oxidative stress and activation of Akt‐eNOS signaling. Am J Physiol Heart Circ Physiol 299: H1419–1427. 10.1152/ajpheart.00591.2010. 20729399 PMC2993212

[aos14954-bib-0210] Shen B , Hagiwara M , Yao YY , Chao L & Chao J (2008): Salutary effect of kallistatin in salt‐induced renal injury, inflammation, and fibrosis via antioxidative stress. Hypertension 51: 1358–1365. 10.1161/hypertensionaha.107.108514. 18391098

[aos14954-bib-0211] Shimizu E , Funatsu H , Yamashita H , Yamashita T & Hori S (2002): Plasma level of interleukin‐6 is an indicator for predicting diabetic macular edema. Jpn J Ophthalmol 46: 78–83. Retrieved from http://www.sciencedirect.com/science/article/pii/S002151550100452X. 11853719 10.1016/s0021-5155(01)00452-x

[aos14954-bib-0212] Shukla R , Gudlavalleti MV , Bandyopadhyay S et al. (2016): Perception of care and barriers to treatment in individuals with diabetic retinopathy in India: 11‐city 9‐state study. Indian J Endocrinol Metab 20(Suppl 1): S33–41. 10.4103/2230-8210.179772. 27144135 PMC4847448

[aos14954-bib-0213] Siddiqui MK , Kennedy G , Carr F et al. (2018): Lp‐PLA2 activity is associated with increased risk of diabetic retinopathy: a longitudinal disease progression study. Diabetologia 61: 1344–1353. 10.1007/s00125-018-4601-7. 29623345 PMC6447502

[aos14954-bib-0214] Silva IT , Mello AP & Damasceno NR (2011): Antioxidant and inflammatory aspects of lipoprotein‐associated phospholipase A(2) (Lp‐PLA(2)): a review. Lipids Health Dis 10: 170. 10.1186/1476-511x-10-170. 21955667 PMC3204246

[aos14954-bib-0215] Simo R , Higuera M , Garcia‐Ramirez M , Canals F , Garcia‐Arumi J & Hernandez C (2008): Elevation of apolipoprotein A‐I and apolipoprotein H levels in the vitreous fluid and overexpression in the retina of diabetic patients. Arch Ophthalmol 126: 1076–1081. 10.1001/archopht.126.8.1076. 18695102

[aos14954-bib-0216] Simó R , Stitt AW & Gardner TW (2018): Neurodegeneration in diabetic retinopathy: does it really matter? Diabetologia 61: 1902–1912. 10.1007/s00125-018-4692-1. 30030554 PMC6096638

[aos14954-bib-0217] Sivaprasad S , Raman R , Conroy D et al. (2020): The ORNATE India Project: United Kingdom‐India Research Collaboration to tackle visual impairment due to diabetic retinopathy. Eye 34: 1279–1286. 10.1038/s41433-020-0854-8. 32398841 PMC7314790

[aos14954-bib-0218] Skelly DT , Hennessy E , Dansereau M‐A & Cunningham C (2013): A systematic analysis of the peripheral and CNS effects of systemic LPS, IL‐1β, TNF‐α and IL‐6 challenges in C57BL/6 mice. PLoS One 8: e69123. 10.1371/journal.pone.0069123. 23840908 PMC3698075

[aos14954-bib-0219] Sohn EH , van Dijk HW , Jiao C et al. (2016): Retinal neurodegeneration may precede microvascular changes characteristic of diabetic retinopathy in diabetes mellitus. Proc Natl Acad Sci USA 113: E2655–2664. 10.1073/pnas.1522014113. 27114552 PMC4868487

[aos14954-bib-0220] Somani R , Richardson VR , Standeven KF , Grant PJ & Carter AM (2012): Elevated properdin and enhanced complement activation in first‐degree relatives of South Asian subjects with type 2 diabetes. Diabetes Care 35: 894–899. 10.2337/dc11-1483. 22338105 PMC3308267

[aos14954-bib-0221] Son JW , Jang EH , Kim MK et al. (2011): Diabetic retinopathy is associated with subclinical atherosclerosis in newly diagnosed type 2 diabetes mellitus. Diabetes Res Clin Pract 91: 253–259. 10.1016/j.diabres.2010.11.005. 21129801

[aos14954-bib-0222] Stafforini DM , Elstad MR , McIntyre TM , Zimmerman GA & Prescott SM (1990): Human macrophages secret platelet‐activating factor acetylhydrolase. J Biol Chem 265: 9682–9687. 2351664

[aos14954-bib-0223] Staurenghi G , Ye L , Magee MH , Danis RP , Wurzelmann J , Adamson P & McLaughlin MM (2015): Darapladib, a lipoprotein‐associated phospholipase A2 inhibitor, in diabetic macular edema: a 3‐month placebo‐controlled study. Ophthalmology 122: 990–996. 10.1016/j.ophtha.2014.12.014. 25749297

[aos14954-bib-0224] Stitt AW (2010): AGEs and diabetic retinopathy. Invest Ophthalmol Vis Sci 51: 4867–4874. 10.1167/iovs.10-5881. 20876889

[aos14954-bib-0225] Suguro T , Watanabe T , Kodate S , Xu G , Hirano T , Adachi M & Miyazaki A (2008): Increased plasma urotensin‐II levels are associated with diabetic retinopathy and carotid atherosclerosis in Type 2 diabetes. Clin Sci 115: 327–334. 10.1042/cs20080014. 18338983

[aos14954-bib-0226] Sun WY , Witte DP , Degen JL et al. (1998): Prothrombin deficiency results in embryonic and neonatal lethality in mice. Proc Natl Acad Sci USA 95: 7597–7602. 10.1073/pnas.95.13.7597. 9636195 PMC22695

[aos14954-bib-0227] Sundaram M & Yao Z (2012): Intrahepatic role of exchangeable apolipoproteins in lipoprotein assembly and secretion. Arterioscler Thromb Vasc Biol 32: 1073–1078. 10.1161/ATVBAHA.111.241455. 22517365

[aos14954-bib-0228] Takada T , Kodera Y , Matsubara M , Kawashima Y , Maeda T , Fujita Y & Shichiri MJA (2013): Serum monomeric α2‐macroglobulin as a clinical biomarker in diabetes. Atherosclerosis 228: 270–276. 23535567 10.1016/j.atherosclerosis.2013.02.035

[aos14954-bib-0229] Takahashi H , Tampo H , Arai Y , Inoue Y & Kawashima H (2017): Applying artificial intelligence to disease staging: Deep learning for improved staging of diabetic retinopathy. PLoS One 12: e0179790. 10.1371/journal.pone.0179790. 28640840 PMC5480986

[aos14954-bib-0230] Takebayashi K , Suetsugu M , Wakabayashi S , Aso Y & Inukai T (2007): Retinol binding protein‐4 levels and clinical features of type 2 diabetes patients. J Clin Endocrinol Metab 92: 2712–2719. 10.1210/jc.2006-1249. 17440021

[aos14954-bib-0231] Thompson A , Gao P , Orfei L et al. (2010): Lipoprotein‐associated phospholipase A(2) and risk of coronary disease, stroke, and mortality: collaborative analysis of 32 prospective studies. Lancet 375: 1536–1544. 10.1016/s0140-6736(10)60319-4. 20435228 PMC2864403

[aos14954-bib-0232] Ting DSW , Cheung CY‐L , Lim G et al. (2017): Development and validation of a deep learning system for diabetic retinopathy and related eye diseases using retinal images from multiethnic populations with diabetes. JAMA 318: 2211–2223. 10.1001/jama.2017.18152. 29234807 PMC5820739

[aos14954-bib-0233] Ting DSW , Tan K‐A , Phua V , Tan GSW , Wong CW & Wong TY (2016): Biomarkers of diabetic retinopathy. Curr Diab Rep 16: 125. 10.1007/s11892-016-0812-9. 27778251

[aos14954-bib-0234] Trucco E , Ruggeri A , Karnowski T et al. (2013): Validating retinal fundus image analysis algorithms: issues and a proposal. Invest Ophthalmol Vis Sci 54: 3546–3559. 10.1167/iovs.12-10347. 23794433 PMC4597487

[aos14954-bib-0235] Tufail A , Rudisill C , Egan C et al. (2017): Automated diabetic retinopathy image assessment software: diagnostic accuracy and cost‐effectiveness compared with human graders. Ophthalmology 124: 343–351. 10.1016/j.ophtha.2016.11.014. 28024825

[aos14954-bib-0236] Turecký L , Kupcova V , & Szantova MJBII (1999): Alpha 2‐macroglobulin in the blood of patients with diabetes mellitus. Bratisl Lek Listy 100: 25–27. 10492994

[aos14954-bib-0237] Turk V , Stoka V & Turk D (2008): Cystatins: biochemical and structural properties, and medical relevance. Front Biosci 13: 5406–5420. 18508595 10.2741/3089

[aos14954-bib-0238] Vo‐Dinh T (2008): Nanosensing at the single cell level. Spectrochim Acta, Part B 63: 95–103. 10.1016/j.sab.2007.11.027 PMC4022309 24839348

[aos14954-bib-0239] Voegele AF , Jerkovic L , Wellenzohn B , Eller P , Kronenberg F , Liedl KR & Dieplinger H (2002): Characterization of the vitamin E‐binding properties of human plasma afamin. Biochemistry 41: 14532–14538. 12463752 10.1021/bi026513v

[aos14954-bib-0240] Vujosevic S , Aldington SJ , Silva P , Hernández C , Scanlon P , Peto T & Simó R (2020): Screening for diabetic retinopathy: new perspectives and challenges. Lancet Diabetes Endocrinol 8: 337–347. 10.1016/S2213-8587(19)30411-5. 32113513

[aos14954-bib-0241] Vujosevic S , Benetti E , Massignan F , Pilotto E , Varano M , Cavarzeran F , Avogaro A & Midena E (2009): Screening for diabetic retinopathy: 1 and 3 nonmydriatic 45‐degree digital fundus photographs vs 7 standard early treatment diabetic retinopathy study fields. Am J Ophthalmol 148: 111–118. 10.1016/j.ajo.2009.02.031. 19406376

[aos14954-bib-0242] Walport MJ (2001a): Complement. First of two parts. N Engl J Med 344: 1058–1066. 10.1056/nejm200104053441406. 11287977

[aos14954-bib-0243] Walport MJ (2001b): Complement. Second of two parts. N Engl J Med 344: 1140–1144. 10.1056/nejm200104123441506. 11297706

[aos14954-bib-0244] Wang J , Yang MM , Li YB , Liu GD , Teng Y & Liu XM (2013a): Association of CFH and CFB gene polymorphisms with retinopathy in type 2 diabetic patients. Mediators Inflamm 2013: 748435. 10.1155/2013/748435. 23864767 PMC3707223

[aos14954-bib-0245] Wang X , Abraham S , McKenzie JAG et al. (2013b): LRG1 promotes angiogenesis by modulating endothelial TGF‐beta signalling. Nature 499: 306–311. 10.1038/nature12345. 23868260 PMC3836402

[aos14954-bib-0246] Wang X , Schmidt DR , Joyce EJ , Kao WJJJoBS & Polymer Edition . (2011): Application of MS‐based proteomics to study serum protein adsorption/absorption and complement C3 activation on poly (ethylene glycol) Hydrogels. J Biomater Sci Polym Ed 22: 1343–1362. 20594411 10.1163/092050610X508400 PMC3855078

[aos14954-bib-0247] Wautier MP , Massin P , Guillausseau PJ , Huijberts M , Levy B , Boulanger E , Laloi‐Michelin M & Wautier JL (2003): N(carboxymethyl)lysine as a biomarker for microvascular complications in type 2 diabetic patients. Diabetes Metab 29: 44–52. 10.1016/S1262-3636(07)70006-X. 12629447

[aos14954-bib-0248] Weivoda S , Andersen JD , Skogen A et al. (2008): ELISA for human serum leucine‐rich alpha‐2‐glycoprotein‐1 employing cytochrome c as the capturing ligand. J Immunol Methods 336: 22–29. 18436231 10.1016/j.jim.2008.03.004 PMC7094546

[aos14954-bib-0249] Wells JA , Glassman AR , Ayala AR et al. (2016): Aflibercept, bevacizumab, or ranibizumab for diabetic macular edema: two‐year results from a comparative effectiveness randomized clinical trial. Ophthalmology 123: 1351–1359. 10.1016/j.ophtha.2016.02.022. 26935357 PMC4877252

[aos14954-bib-0250] Wilkinson CP , Ferris FL III , Klein RE et al. (2003): Proposed international clinical diabetic retinopathy and diabetic macular edema disease severity scales. Ophthalmology 110: 1677–1682. 10.1016/S0161-6420(03)00475-5. 13129861

[aos14954-bib-0251] Williams GA , Scott IU , Haller JA , Maguire AM , Marcus D & McDonald HR (2004): Single‐field fundus photography for diabetic retinopathy screening: a report by the American Academy of Ophthalmology. Ophthalmology 111: 1055–1062. 10.1016/j.ophtha.2004.02.004. 15121388

[aos14954-bib-0252] Wong CW , Teo BW , Lamoureux E et al. (2015): Serum cystatin C, markers of chronic kidney disease, and retinopathy in persons with diabetes. J Diabetes Res 2015: 404280. 10.1155/2015/404280. 26576434 PMC4630396

[aos14954-bib-0253] Wong TY , Mwamburi M , Klein R et al. (2009): Rates of progression in diabetic retinopathy during different time periods: a systematic review and meta‐analysis. Diabetes Care 32: 2307–2313. 10.2337/dc09-0615. 19940227 PMC2782996

[aos14954-bib-0254] Woo C‐H , Eom Y‐W , Yoo M‐H et al. (2000): Tumor necrosis factor‐α generates reactive oxygen species via a cytosolic phospholipase A2‐linked cascade. J Biol Chem 275: 32357–32362. 10.1074/jbc.M005638200. 10934206

[aos14954-bib-0255] Wu M‐Y , Yiang G‐T , Lai T‐T & Li C‐J (2018): The oxidative stress and mitochondrial dysfunction during the pathogenesis of diabetic retinopathy. Oxid Med Cell Longevity 2018: 12. 10.1155/2018/3420187. PMC6145164 30254714

[aos14954-bib-0256] Xu J , Chen LJ , Yu J , Wang HJ , Zhang F , Liu Q & Wu J (2018): Involvement of advanced glycation end products in the pathogenesis of diabetic retinopathy. Cell Physiol Biochem 48: 705–717. 10.1159/000491897. 30025404

[aos14954-bib-0257] Yagame M , Suzuki D , Jinde K et al. (1997): Significance of urinary type IV collagen in patients with diabetic nephropathy using a highly sensitive one‐step sandwich enzyme immunoassay. J Clin Lab Anal 11: 110–116. 10.1002/(sici)1098-2825(1997)11:2<110:Aid-jcla7>3.0.Co;2-f. 9058245 PMC6760738

[aos14954-bib-0258] Yang W , Lu J , Weng J et al. (2010): Prevalence of diabetes among men and women in China. N Engl J Med 362: 1090–1101. 10.1056/NEJMoa0908292. 20335585

[aos14954-bib-0259] Yao Y , Li R , Du J , Long L , Li X & Luo N (2019): Interleukin‐6 and diabetic retinopathy: a systematic review and meta‐analysis. Curr Eye Res 44: 564–574. 10.1080/02713683.2019.1570274. 30644770

[aos14954-bib-0260] Yasuhara O , Hanai K , Ohkubo I , Sasaki M , McGeer PL & Kimura H (1993): Expression of cystatin C in rat, monkey and human brains. Brain Res 628(1–2): 85–92. 8313175 10.1016/0006-8993(93)90941-f

[aos14954-bib-0261] Yau JW , Rogers SL , Kawasaki R et al. (2012): Global prevalence and major risk factors of diabetic retinopathy. Diabetes Care 35: 556–564. 10.2337/dc11-1909. 22301125 PMC3322721

[aos14954-bib-0262] Yin H , Gao L , Shen B , Chao L & Chao J (2010): Kallistatin inhibits vascular inflammation by antagonizing tumor necrosis factor‐alpha‐induced nuclear factor kappaB activation. Hypertension 56: 260–267. 10.1161/hypertensionaha.110.152330. 20566960 PMC2922869

[aos14954-bib-0263] Yoshino S , Fujimoto K , Takada T , Kawamura S , Ogawa J , Kamata Y , Kodera Y & Shichiri M (2019): Molecular form and concentration of serum α(2)‐macroglobulin in diabetes. Sci Rep 9: 12927. 10.1038/s41598-019-49144-7. 31506491 PMC6736885

[aos14954-bib-0264] Youngblood H , Robinson R , Sharma A & Sharma S (2019): Proteomic biomarkers of retinal inflammation in diabetic retinopathy. Int J Mol Sci 20: 4755. Retrieved from https://www.mdpi.com/1422‐0067/20/19/4755. 31557880 10.3390/ijms20194755 PMC6801709

[aos14954-bib-0265] Yui Y , Aoyama T , Morishita H , Takahashi M , Takatsu Y & Kawai C (1988): Serum prostacyclin stabilizing factor is identical to apolipoprotein A‐I (Apo A‐I). A novel function of Apo A‐I. J Clin Invest 82: 803–807. 10.1172/jci113682. 3047170 PMC303586

[aos14954-bib-0266] Zabetian‐Targhi F , Mahmoudi MJ , Rezaei N & Mahmoudi M (2015): Retinol binding protein 4 in relation to diet, inflammation, immunity, and cardiovascular diseases. Adv Nutr 6: 748–762. 10.3945/an.115.008292. 26567199 PMC4642414

[aos14954-bib-0267] Zhang C , Li K , Zhang J , Kuang X , Liu C , Deng Q & Li D (2019a): Relationship between retinol and risk of diabetic retinopathy:a case‐control study. Asia Pac J Clin Nut 28: 607–613. 10.6133/apjcn.201909_28(3).0021. 31464408

[aos14954-bib-0268] Zhang J , Zhu L , Fang J , Ge Z & Li X (2016): LRG1 modulates epithelial‐mesenchymal transition and angiogenesis in colorectal cancer via HIF‐1α activation. J Exp Clin Cancer Res 35: 29. 26856989 10.1186/s13046-016-0306-2 PMC4746930

[aos14954-bib-0269] Zhang Q , Hu J , Hu Y , Ding Y , Zhu J & Zhuang C (2018): Relationship between serum apolipoproteins levels and retinopathy risk in subjects with type 2 diabetes mellitus. Acta Diabetol 55: 681–689. 10.1007/s00592-018-1136-9. 29623430

[aos14954-bib-0270] Zhang X , Pek SLT , Tavintharan S et al. (2019b): Leucine‐rich α‐2‐glycoprotein predicts proliferative diabetic retinopathy in type 2 diabetes. J Diabetes Complications. 10.1016/j.jdiacomp.2019.05.021. 31256924

[aos14954-bib-0271] Zhang Y , Sun X , Icli B & Feinberg MW (2017): Emerging roles for MicroRNAs in diabetic microvascular disease: novel targets for therapy. Endocrine Rev 38: 145–168. 10.1210/er.2016-1122. 28323921 PMC5460677

[aos14954-bib-0272] Zhou B , Lu Y , Hajifathalian K et al. (2016): Worldwide trends in diabetes since 1980: a pooled analysis of 751 population‐based studies with 4·4 million participants. Lancet 387: 1513–1530. 10.1016/S0140-6736(16)00618-8. 27061677 PMC5081106

[aos14954-bib-0273] Zhou G , Chao L , Chao JJJoBC (1992): Kallistatin: a novel human tissue kallikrein inhibitor. Purification, characterization, and reactive center sequence. J Biol Chem 267: 25873–25880. 1334488

[aos14954-bib-0274] Zhou Z , Ju H , Sun M & Chen H (2019): Serum vascular endothelial growth factor levels correlate with severity of retinopathy in diabetic patients: a systematic review and meta‐analysis. Dis Markers 2019: 15. 10.1155/2019/9401628. PMC6451802 31019585

[aos14954-bib-0275] Zhu X‐R , Yang F‐Y , Lu J , Zhang H‐R , Sun R , Zhou J‐B & Yang J‐K (2019): Plasma metabolomic profiling of proliferative diabetic retinopathy. Nutr Metab 16: 37. 10.1186/s12986-019-0358-3. PMC6540396 31160916

[aos14954-bib-0276] Zorena K , Myśliwska J & Myśliwiec M et al. (2007): Serum TNF‐alpha level predicts nonproliferative diabetic retinopathy in children. Mediators Inflamm 2007: 5. 10.1155/2007/92196. PMC1906713 17641733

[aos14954-bib-0277] Zurdel J , Finckh U , Menzer G , Nitsch RM & Richard G (2002): *CST3* genotype associated with exudative age related macular degeneration. Br J Ophthalmol 86: 214–219. 10.1136/bjo.86.2.214. 11815350 PMC1771004

